# Tripterhyponoid A from *Tripterygium hypoglaucum* Inhibiting MRSA by Multiple Mechanisms

**DOI:** 10.3390/molecules30122539

**Published:** 2025-06-10

**Authors:** Yan-Yan Zhu, Qiong Jin, Zhao-Jie Wang, Mei-Zhen Wei, Wen-Biao Zu, Zhong-Shun Zhou, Bin-Yuan Hu, Yun-Li Zhao, Xu-Jie Qin, Xiao-Dong Luo

**Affiliations:** 1 Yunnan Characteristic Plant Extraction Laboratory, Key Laboratory of Medicinal Chemistry for Natural Resource, Ministry of Education and Yunnan Province, School of Chemical Science and Technology, Yunnan University, Kunming 650500, China; 22021016018@mail.ynu.edu.cn (Y.-Y.Z.); wzhj@mail.ynu.edu.cn (Z.-J.W.); 22021016017@mail.ynu.edu (M.-Z.W.); wenbiao-zu@foxmail.com (W.-B.Z.); zhouzhongshun@mail.ynu.edu.cn (Z.-S.Z.); hubinyuan2018@163.com (B.-Y.H.); zhaoyunli@mail.kib.ac.cn (Y.-L.Z.); 2 State Key Laboratory of Phytochemistry and Plant Resources in West China, Kunming Institute of Botany, Chinese Academy of Sciences, Kunming 650201, China; jinqiong@mail.kib.ac.cn (Q.J.); qinxujie@mail.kib.ac.cn (X.-J.Q.)

**Keywords:** tripterhyponoid A, anti-MRSA, quorum sensing, antibiofilm, DNA binding

## Abstract

The emergence of methicillin-resistant *Staphylococcus aureus* (MRSA) and its biofilm-forming ability underscore the limitations of current antibiotics. In this study, a new compound named tripterhyponoid A was found to effectively combat MRSA, with an MIC of 2.0 μg/mL. It inhibited biofilm formation by downregulating genes related to the quorum sensing (QS) pathway (*sarA*, *agrA*, *agrB*, *agrC*, *agrD*, and *hld*) and eradicated mature biofilms. Furthermore, it induced DNA damage by binding to bacterial DNA, enhancing its efficiency against MRSA. Therefore, its anti-MRSA properties with multiple mechanisms of action make it less prone to developing resistance over 20 days. In addition, it reduced the bacterial load and regulated the levels of inflammatory cytokines IL-6 and IL-10 at the wound site in a mouse skin infection model. This paper provides the first in-depth investigation of the mechanisms of triterpenoids against MRSA by inhibiting the expression of QS system genes and binding to DNA.

## 1. Introduction

The abuse of antibiotics has caused the emergence of multi-drug-resistant (MDR) bacteria, such as methicillin-resistant *Staphylococcus aureus* (MRSA), leading to a spectrum of infections that range from minor skin disorders to serious illnesses like sepsis, meningitis, pneumonia, and osteomyelitis [1]. An analysis of the 1990–2021 global bacterial antimicrobial resistance burden predicted that over 39 million people might die from infections caused by bacteria antimicrobial resistance (AMR) within the next 25 years. The number of deaths caused by MRSA resulted in 130,000 fatalities directly, more than doubling the 57,200 deaths recorded in 1990 [2].

Bacteria produce extracellular DNA (eDNA), proteins, and polysaccharides to construct a biofilm matrix. This matrix functions as a structural scaffold for the three-dimensional architecture of the biofilm and confers protection against diverse environmental challenges, such as antibiotics and host immune responses [3,4]. Quorum sensing (QS) is a crucial mechanism that regulates biofilm formation and virulence in *S. aureus*. *S. aureus* employs QS molecules to facilitate communication, discern the cell density, and respond accordingly. The QS system regulates various physiological processes in bacteria, including biofilm formation, the majority of virulence factors, secondary metabolite production, and motility, which is closely associated with antibiotic resistance in these microorganisms [3,5]. The development of biofilms represents a significant virulence factor in MRSA, contributing to chronic persistent infections or severe cases that often result in the failure of antibiotic treatments [3,6]. Collectively, biofilm formation results in antibiotic resistance that is 10 to 1000 times greater than that of corresponding planktonic bacteria, which accounts for the persistence and recurrence of such infections [7]. Therefore, the discovery of antibacterial compounds with biofilm destruction abilities is crucial in addressing MRSA infections [8].

Unlike mammals, plants lack a complex immune system to defend against microbial invasion, so their secondary metabolites might become a crucial source of antimicrobial compounds [9]. Recent studies have shown that triterpenoids hold considerable therapeutic potential in reducing planktonic bacteria and combating biofilms, yet certain limitations remain. For instance, while betulinic acid has demonstrated activity against the interkingdom biofilm *S. aureus*–*Candida albicans*, it cannot kill microorganisms [10]. Similarly, camellia saponins can inhibit the growth of *Bacillus cereus* and prevent its biofilm formation; however, an effective concentration as high as 64 mg/mL is required [11], which may limit its practical application. In our ongoing quest for natural plant products that are effective against drug-resistant bacteria and fungi, we have investigated flavonoids [12,13,14], terpenoids [15], alkaloids [16,17], steroids [18], and polyphenols [19], which operate through various mechanisms and pathways. *Tripterygium hypoglaucum* (H.Lév.) Hutch is a toxic vine that has been utilized in traditional Chinese medicine for the treatment of inflammatory and autoimmune disorders [20,21]; however, there is limited research on its antibacterial properties. To date, approximately 300 compounds have been isolated, mainly terpenoids and alkaloids, of which terpenoids account for more than 50%, with triterpenoids accounting for roughly 20% and representing one of the main types of active components [22,23]. In this study, we found a new triterpenoid, tripterhyponoid A, from *T. hypoglaucum*, which demonstrated significant antibacterial and antibiofilm activity against MRSA. The objectives of this study were to investigate the antibacterial and antibiofilm activity of tripterhyponoid A and elucidate the underlying mechanisms involved.

## 2. Results and Discussion

### 2.1. New Structure Elucidation

**Figure 1:**
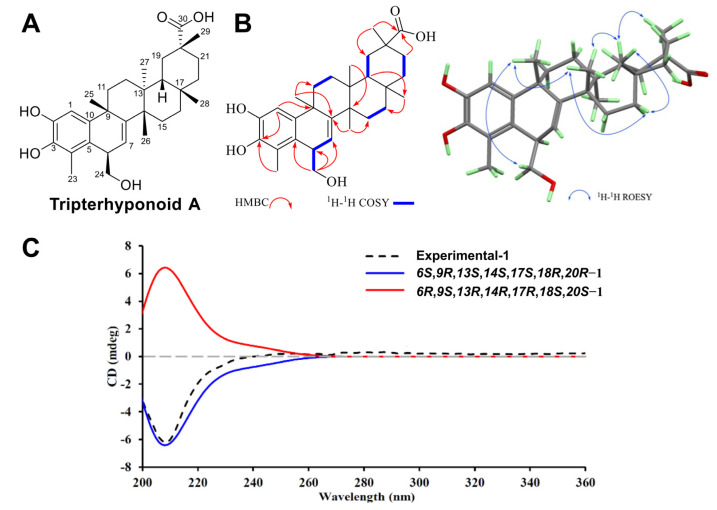
Structure elucidation of tripterhyponoid A. (**A**) Chemical structure. (**B**) Key correlations of ^1^H-^1^H COSY, HMBC, and ROESY spectra. (**C**) Experimental and calculated CD spectra.

The molecular formula of the new compound was C_30_H_42_O_5_ based on the ion peak at *m/z* 481.2953 [M − H]^−^ (calcd. for 481.2959) of HRESIMS, which indicated 10 degrees of unsaturation. Its UV spectrum showed maximal absorption bands at 203 and 426 nm, suggesting the existence of an aromatic ring. Its IR spectrum suggested the presence of hydroxyl (3439 cm^−1^), carboxyl (1639 cm^−1^), and aromatic ring (1449 cm^−1^) functional groups [24]. Its ^1^H NMR spectrum showed six methyl groups [*δ*_H_ 2.21 (H-23), 1.47 (H-25), 1.28 (H-26), 0.75 (H-27), 1.10 (H-28), 1.17 (H-29)] and two aromatic ring protons [*δ*_H_ 6.69 (s, H-1), 6.02 (H-7, d, *J* = 6.1 Hz)] (Table 1). In its ^13^C NMR and DEPT spectra, 30 carbon signals were observed and classified into eight methylene groups (*δ*_C_ 37.1, 31.6, 30.1, 38.1, 30.9, 31.7, 36.0, 69.2), four methine groups (*δ*_C_ 110.0, 42.0, 122.7, 45.9), 6 sp^2^ nonprotonated carbons (*δ*_C_ 142.2, 142.9, 121.8, 125.2, 151.2, 144.7), and one carboxyl group carbon (*δ*_C_ 182.8). Detailed spectral data analysis suggested a friedelane similar to triptohypol C [25], with an additional hydroxymethyl group at C-6. This assumption was further supported by the key correlation of *δ*_H_ 3.71 (H-24) with *δ*_C_ 42.0 (C-6), 125.2 (C-5), and 122.6 (C-7) and of *δ*_H_ 3.58 (H-6) with *δ*_C_ 69.2 (C-24), 125.2 (C-5), 122.6 (C-7), 121.8 (C-4), and 142.9 (C-10) in its HMBC spectrum. Additionally, the correlations among *δ*_H_ 6.02 (H-7)/3.58 (H-6)/3.71 (H-24) in the ^1^H-^1^H COSY spectrum provided supporting evidence (Figure 1B). The configuration of C-25 was regarded as a *β*-orientation biogenetically [26,27]. The relative configuration was determined by its ROESY spectrum (Figure 1B), with NOE correlations of *δ*_H_ 3.26 (H-24)/1.47 (H-25)/1.28 (H-26)/1.58 (H-18)/1.17 (H-29) and of *δ*_H_ 1.26 (H-26)/1.90 (H-16)/1.10 (H-28) indicating the *β*-orientations of all these protons. Subsequently, the absolute configuration was deduced to be 6*S*, 9*R*, 13*S*, 14*S*, 17*S*, 18*R*, 20*R* based on the well-matched experimental and calculated ECD spectra (Figure 1C), and it was then named tripterhyponoid A (Figure 1A).

### 2.2. Tripterhyponoid A Significantly Inhibited MRSA In Vitro

**Figure 2:**
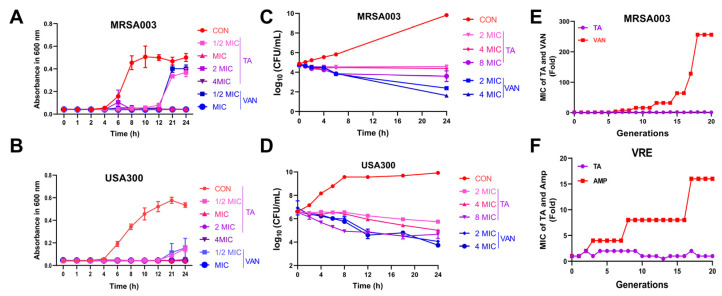
Antibacterial activity of tripterhyponoid A in vitro. (**A**,**B**) Growth curves against MRSA003 and USA300. (**C**,**D**) Time-kill curves against MRSA003 and USA300. (**E**,**F**) Development of resistance in MRSA003 and VRE. Values indicating fold changes in MIC relative to the MIC of the initial passage. TA, tripterhyponoid A; VAN, vancomycin hydrochloride.

To evaluate the antibacterial activity of tripterhyponoid A, we determined its MIC against Gram-negative (G−) and Gram-positive (G+) bacteria. It exhibited broad-spectrum and significant antibacterial activity against various G+ bacteria, with an MIC range of 1 to 8 μg/mL (specifically, 2 μg/mL for MRSA and 4 μg/mL for VRE). Compared to many antibiotics, its antibacterial efficacy is notably superior. For example, the MIC range of ampicillin sodium (AMP) for MRSA is as high as 128–512 μg/mL, while the MIC of vancomycin hydrochloride (VAN) for VRE is 32 μg/mL. We also confirmed that tripterhyponoid A did not exhibit antibacterial activity against G− bacteria at the evaluated concentrations (MICs > 16 μg/mL) (Table S1) [28]. We subsequently evaluated the growth-inhibitory effects of tripterhyponoid A against MRSA and VRE. It prolonged the logarithmic phase, increased the time taken for bacteria to reach the stationary phase at a concentration of 1/2 × MIC, and completely inhibited the growth of the strains at concentrations of 1–4 × MIC (Figure 2A,B and Figure S11A). Furthermore, it exhibited minimal bactericidal effects at 8 × MIC, while significant bacteriostatic effects were observed at 2 × and 4 × MIC (Figure 2C,D and Figure S11B) [29,30]. In line with the research by Chen et al., tripterhyponoid A maintained its effectiveness against MRSA throughout the experiment (Figure 2E,F), unlike the tested antibiotics (AMP and VAN), which induced the resistance within 20 generations. This highlights its potent efficacy [31].

### 2.3. Exploration of Anti-MRSA Pathways Through Transcriptomics Analysis

**Figure 3:**
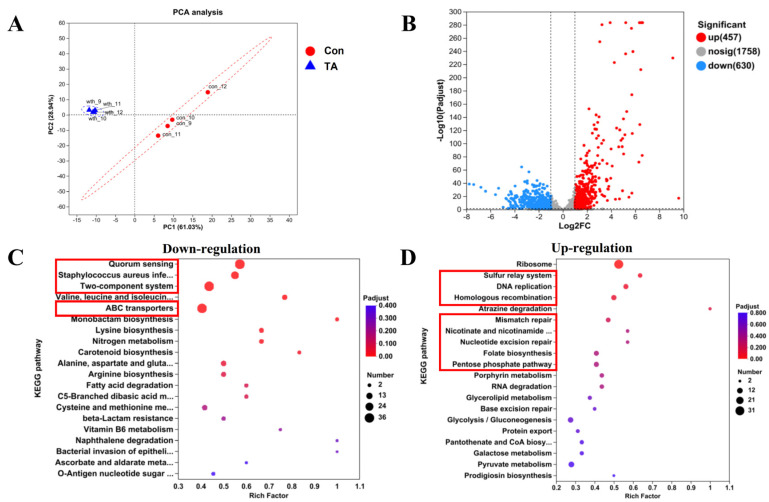
Transcriptomics analysis of USA300 treated by tripterhyponoid A. (**A**) Principal component analysis. (**B**) The distribution of differentially expressed genes (DEGs) in a volcano plot, with red dots representing upregulated genes and blue dots representing downregulated genes. (**C**,**D**) KEGG enrichment analysis for downregulated and upregulated genes, respectively. Con, DMSO; TA, tripterhyponoid A.

To explore the potential pathways of tripterhyponoid A regarding anti-MRSA activity, transcriptomics analysis was used. A total of 1087 differentially expressed genes (DEGs) were identified, including 457 upregulated and 630 downregulated genes, exhibiting highly significant expression patterns (|log2 (fold change) | > 1, FDR < 0.05). The transcriptomics analysis revealed highly significant expression differences before and after tripterhyponoid A treatment (Figure 3A,B) [29]. Kyoto Encyclopedia of Genes and Genomes (KEGG) and Gene Ontology (GO) analyses indicated that virulence-related pathways, including QS systems, two-component systems (TCS), and carotenoid biosynthesis, as well as energy metabolism pathways such as ATP-binding cassette (ABC) transport and multiple amino acid synthesis and metabolic processes, were downregulated (Figure 3C and Figure S12A). Conversely, pathways associated with ribosome function and DNA replication and repair were upregulated (Figure 3D and Figure S12B). The transcriptomics analysis showed that tripterhyponoid A disrupted the expression of genes associated with *S. aureus* biofilm formation and virulence factors, while simultaneously activating DNA replication and repair pathways [32,33].

### 2.4. Tripterhyponoid A Repressed the Expression of Biofilm- and Virulence-Associated Genes

**Figure 4:**
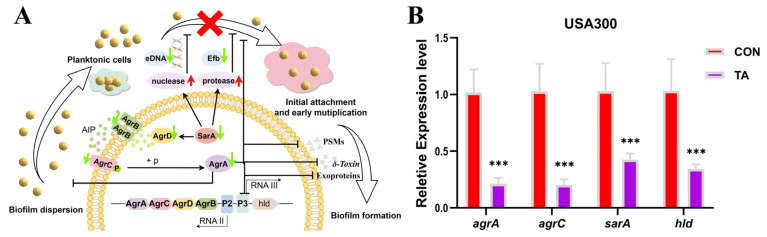
Antibiofilm mechanism of tripterhyponoid A (TA) against MRSA. (**A**) Model of agr-QS system inhibition mechanism (by FigDraw). The green arrows indicate genes downregulated by TA. (**B**) The RT-qPCR analysis of genes involved in the QS system. Values are mean ± SD (n = 3). *** *p* < 0.001 vs. CON.

We conducted a comparative analysis of our list of DEGs in relation to previously reported genes to elucidate the molecular mechanisms through which tripterhyponoid A inhibits biofilm formation and virulence. Our findings indicated that numerous genes encoding virulence factors, surface proteins, and capsular polysaccharides related to the biofilm formation of the USA300 strain were significantly downregulated under treatment with tripterhyponoid A (Table S2), which was consistent with the results of Bhawandeep et al. [34]. Notably, the levels of genes associated with the QS system, namely *sarA*, *agrA*, *agrB*, *agrC*, and *agrD*, in the samples were decreased 2.19-, 3.65-, 3.65-, 3.69-, and 4.16-fold, respectively (Figure 4A and Figure S13A). Additionally, genes (*hld*) encoding virulence factors (*δ*-hemolysin) were downregulated, with log2 FC = −3.7 and −3.2, respectively (Table S2). *SarA*, an essential global regulator, interacts with target promoters to influence the expression of multiple virulence factors in *S. aureus*. It is necessary for the effective transcription of RNAII and RNAIII within the accessory gene regulator (*agr*) locus. It has been reported in the literature that the increasing protease and nuclease activity of *sarA* mutants might reduce extracellular fibrinogen binding (Efb) and eDNA release, thereby inhibiting biofilm formation [3,35,36,37]. Phenol-soluble modulins (PSMs) are key virulence factors produced by *S. aureus* that contribute to the formation of biofilms and the spread of biofilm-related infections [38]. Thus, tripterhyponoid A inhibited the *sarA* and QS system, leading to a reduction in the secretion of eDNA, Efb, and PSMs, which might ultimately cause an insufficient supply of essential components for biofilm formation and the attenuation of virulence factors. Furthermore, the genes (*clfA*, *sdrC*, *sdrD*, *fnbA*, *fnbB*, *arlS*, and *arlR*) responsible for cell adhesion and bacterial attachment in staphylococcal biofilm formation were downregulated by tripterhyponoid A (Table S2) [1,39,40,41,42]. In addition, genes related to metabolism and capsular polysaccharide synthesis proteins were highly downregulated in tripterhyponoid A treatment samples (Table S2). The reduction in the synthesis of such components also suggested the decreased production of extracellular substances [42,43]. Then, six functional genes associated with biofilm formation were selected and subsequently subjected to RT-qPCR validation [44]. As a result, the expression levels of *agrA*, *agrC*, *sarA*, and *hld* in tripterhyponoid A-treated USA300 samples exhibited 4.8-, 5.1-, 2.4-, and 3.6-fold reductions, respectively (Figure 4B), and similar results were also observed in MRSA003 samples (Figure S13B). In summary, tripterhyponoid A impeded the initial attachment and adhesion processes during biofilm formation, and it inhibited biofilm formation by suppressing the expression levels of QS-related genes, surface proteins, and secreted proteins, as well as the synthesis proteins of capsular polysaccharides.

### 2.5. Tripterhyponoid A Inhibited Biofilm Formation and Eradicated Mature Biofilms

**Figure 5:**
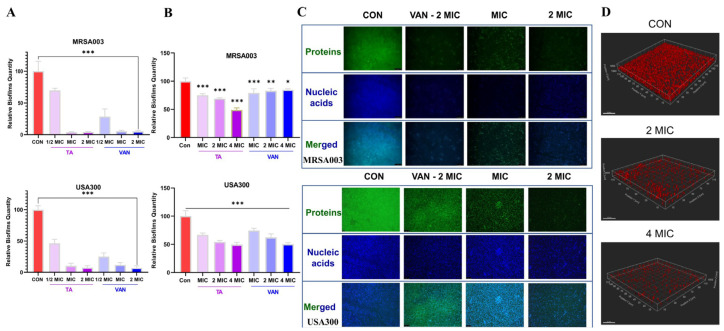
The antibiofilm activity of tripterhyponoid A. (**A**) The inhibitory effect on a MRSA biofilm. (**B**) The elimination effect on a mature MRSA biofilm. (**C**) Fluorescence images of protein and nucleic acid staining for MRSA biofilm disruption. Scale bar: 100 μm. (**D**) The 3D fluorescence confocal micrographs of a USA300 biofilm matrix stained with FilmTracer™ SYPRO^®^ Ruby Biofilm Matrix Stain. Scale bar: 20 μm. Values are mean ± SD (n = 3). * *p* < 0.05, ** *p* < 0.01 and *** *p* < 0.001 vs. CON. CON, DMSO; TA, tripterhyponoid A; VAN, vancomycin hydrochloride.

Crystal violet (CV) staining was employed to evaluate the inhibitory and disruptive effects of tripterhyponoid A on MRSA biofilms [36,45]. As illustrated in Figure 5A, treatment with tripterhyponoid A resulted in negligible MRSA biofilm formation (MRSA003, <5%; USA300, <10%) at the MIC and 2 MIC, comparable to the VAN group. Additionally, it had a significant impact on the biofilm formation of MRSA003 and USA300 at a concentration of 1/2 MIC, with approximate inhibition rates of 30% and 50%, respectively. To evaluate its potential efficacy in eradicating established bacterial biofilms, various concentrations of tripterhyponoid A were applied to 24-h-old bacterial biofilms for scavenging purposes [46]. As depicted in Figure 5B, a concentration of 4 MIC tripterhyponoid A achieved clearance rates of 52% and 51% (*p* < 0.001) for mature biofilms of MRSA003 and USA300. As the concentration of tripterhyponoid A was reduced to 2 MIC and the MIC, the clearance rates correspondingly decreased to 32% and 26% (*p* < 0.001) for MRSA003 and 56% and 34% (*p* < 0.001) for USA300. These findings demonstrate the efficacy of TA in clearing mature MRSA biofilms [47,48].

To gain a better understanding of the MRSA biofilm integrity, a 24-h-old biofilm was treated with tripterhyponoid A, and then the biofilm proteins and DNA were stained with fluorescein isothiocyanate (FITC) and Hoechst 33342, respectively. Fluorescence microscopy was then used to capture images of the biofilm [49]. In the untreated group, both strains exhibited conspicuous blue and green fluorescence signals, suggesting that the biofilm remained intact, with substantial content of proteins and DNA, which were tightly bound. Upon treatment with the MIC and 2 MIC of tripterhyponoid A, the fluorescence signals of the bacterial strains were significantly reduced, indicating that the biofilm had been removed and its structural integrity was destroyed (Figure 5C). Concurrently, a confocal laser scanning microscope (CLSM) was employed to examine the degradation of the biofilm matrix and to assess the viability of the preformed MRSA biofilm under tripterhyponoid A treatment [50]. The biofilm matrices in the control groups exhibited a thick and completely red stain, while the red fluorescence of the biofilm in the treatment group was relatively sparse according to the FilmTracer™ SYPRO^®^ Ruby (Thermo Fisher Scientific, Waltham, MA, USA)(Figure 5D). Generally, the removal of mature biofilms is more challenging than inhibiting their formation [51], and tripterhyponoid A serves as a double-edged sword in inhibiting biofilm formation and clearing mature biofilms to penetrate and target bacteria easily [47,48].

### 2.6. Tripterhyponoid A Upregulated the Expression of Genes Associated with DNA Replication and Repair

**Figure 6:**
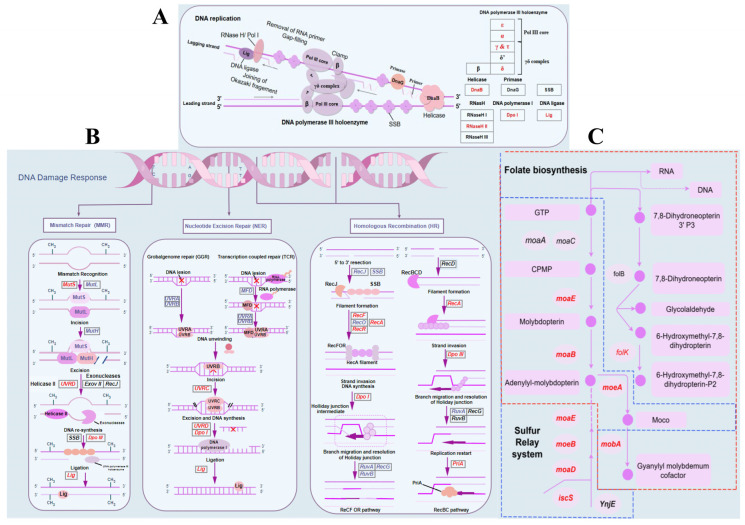
Pathway analysis of DEGs related to DNA replication and repair by tripterhyponoid A (by FigDraw). (**A**) DNA replication pathway. (**B**) DNA damage response pathway. (**C**) Folate biosynthesis and sulfur relay system pathways. Among them, the red text indicates that the DEGs were upregulated.

The transcriptomic sequence analysis indicated that several genes exhibited significant upregulation following treatment with tripterhyponoid A. The top 20 pathways of upregulated genes exhibiting the smallest p adjusted values were selected for partial presentation (Figure 3D). These pathways primarily include the ribosome, the sulfur relay system, DNA replication, homologous recombination, mismatch repair, nucleotide excision repair, folate biosynthesis, and the pentose phosphate pathway (Table S3). The pathways involved in DNA replication and repair are summarized and illustrated in Figure 6. The DNA polymerase III holoenzyme is a multi-subunit protein complex that plays a critical role in the rapid synthesis of DNA in bacteria [52]. Four genes encoding the DNA polymerase III holoenzyme—*dnaE* (α), *dnaQ* (ε), *dnaX* (γ & τ), and *holA* (δ)—during DNA replication were upregulated significantly by tripterhyponoid A according to the transcriptomics analysis (Figure 6A and Figure S14). Meanwhile, the expression levels of DEGs (*folB*, *folK*, *folE2*, *moaB*, *moaE*, and *moeA*) involved in folate biosynthesis and the sulfur relay system were also increased significantly in the treatment group. Furthermore, two DEGs (*moaD* and *iscS*) identified within the sulfur relay system exhibited an upregulatory trend (Figure 6C and Figure S14). In addition, pathways associated with the DNA damage response, including homologous recombination, mismatch repair, and nucleotide excision repair, were also activated by tripterhyponoid A (Figure 6B and Figure S14). External stimuli and stressors frequently compromise the structure and function of DNA, inhibiting its normal replication or causing mutations and, in some cases, even leading to bacterial death. The DNA damage response is capable of rectifying replication errors and addressing specific types of chemical damage to DNA [53]. These findings suggest a deficiency in nucleic acids and DNA lesions in cells under tripterhyponoid A stress to stimulate the upregulation of raw material genes responsible for DNA replication and repair [53].

### 2.7. Tripterhyponoid A Interacted with USA300 Genomic DNA

**Figure 7:**
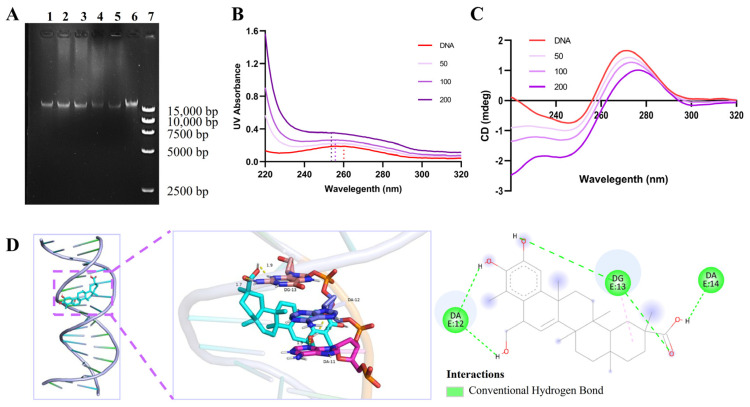
Tripterhyponoid A binds to USA300 genomic DNA. (**A**) Gel retardation analysis. Bands 1–5: tripterhyponoid A/DNA weight ratios of 200/1, 100/1, 50/1, 25/1, and 25/8, respectively; Band 6: untreated DNA, Band 7: marker. (**B**) UV spectra of interaction between tripterhyponoid A and DNA (50 ng/μL). (**C**) CD spectra of DNA in the presence of tripterhyponoid A. (**D**) The complex structure of tripterhyponoid A and DNA (left) and a detailed view of the binding bonds (middle, right).

The binding of compounds to DNA is an effective approach in achieving antibacterial objectives. To elucidate the molecular mechanism of tripterhyponoid A, its effect on genomic DNA was evaluated by analyzing the electrophoretic mobility of DNA bands on an agarose gel [33,54]. The electrophoretogram of the treated group’s DNA (bands 1–5) showed a darkening effect in the DNA bands, suggesting competition between the dye and compound for binding to DNA (Figure 7A). Furthermore, UV–vis spectroscopy was employed to examine the binding interactions between tripterhyponoid A and genomic DNA [55]. As the concentration of tripterhyponoid A increased, the absorption peak of DNA at 260 nm correspondingly intensified, accompanied by a slight blue shift, indicating conformational alterations in the DNA structure (Figure 7B). In addition, the normal DNA displayed positive and negative peaks at approximately 270 nm and 250 nm, but tripterhyponoid A caused a decrease in the ellipticity intensity of the DNA in the CD spectrum (Figure 7C). The results suggest that tripterhyponoid A influences the helical structure of DNA, consistent with the results of the gel assay. Furthermore, *S. aureus*’s DNA structure (4xqq) from the Protein Data Bank (PDB) was selected for docking studies [33,55]; as a result, it was found that tripterhyponoid A might interact with the minor grooves of DNA, which was aligned with the findings from the DNA gel and CD assays. Specifically, the hydroxyl of ring-B in tripterhyponoid A formed two hydrogen bonds with bases DA-11 (1.9 Å) and DA-12 (2.0 Å), and the carbonyl group formed hydrogen bonds with base DG-13 (1.7 Å and 1.9 Å), yielding a binding energy of −9.68 kcal/mol (Figure 7D). Overall, tripterhyponoid A exerted its potent antibacterial activity by inducing bacterial DNA damage through binding to DNA, which significantly affects its efficacy.

### 2.8. Tripterhyponoid A Showed Potential Therapeutic Capabilities in a Mouse Skin Infection Model

**Figure 8:**
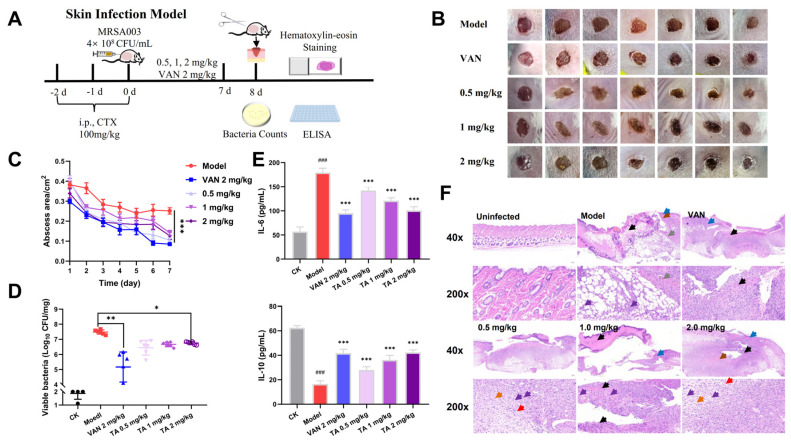
Anti-MRSA activity of tripterhyponoid A (TA) in mouse skin infection model. (**A**) Experimental protocol. (**B**) Representative photographs of wounds infected with MRSA003 after 7 d of treatment. (**C**) Wound sizes after infection with MRSA003 in different groups. (**D**) Bacterial burdens in wounds on the 7th day post-infection. (**E**) Quantification of IL-6 and IL-10 in skin tissue. Model: PBS treatment group; VAN, vancomycin; CTX, cyclophosphamide. ### *p* < 0.001, compared with CK; *** *p* < 0.001, ** *p* < 0.01, or * *p* < 0.05, compared with model group. (**F**) Histological evaluation of mouse skin tissue at different magnifications. Skin tissue necrosis (black arrow), subcutaneous bleeding (grey arrow), granulocyte and lymphocyte infiltration (purple arrow), epidermal thickening (blue arrow), dermis bleeding (brown arrow), granulation tissue and fibrous connective tissue proliferation (orange arrow), new blood vessels (red arrow). Original magnification ×40 or ×200.

*S. aureus* is a common reason for chronic wound infections, so a mouse skin infection model was constructed to evaluate its antibacterial efficacy in vivo (Figure 8A) [6]. The wounds in the VAN and tripterhyponoid A treatment groups showed varying degrees of recovery when compared to the model group, with a significant reduction in the wound size (Figure 8B,C). The results of bacterial counts and H&E staining demonstrated similar trends. In the tripterhyponoid A and VAN treatment groups, the bacterial count at the infectious site exhibited a reduction ranging from 20 to 200 times when compared to the model group (Figure 8D). Histological analysis further corroborated these findings, revealing the significant proliferation of granulation tissue and fibrous connective tissue in the treatment groups, in which the minimal infiltration of granulocytes and lymphocytes and new blood vessels appeared (Figure 8F). Moreover, the low expression of the pro-inflammatory cytokine interleukin-6 (IL-6) and high expression of the anti-inflammatory factor interleukin-10 (IL-10) in the treated groups (Figure 8E) were advantageous in mitigating the inflammatory response. Thus, tripterhyponoid A showed antibacterial properties and the ability to modulate inflammatory factors to reduce the bacterial load and recovery time.

## 3. Materials and Methods

### 3.1. General Experimental Procedures

HRESI-MS analysis was performed with the Agilent 1290 UHPLC/6545 Q-TOF system (Agilent Technologies, Santa Clara, CA, USA). A Bruker AM-500 MHz spectrometer was used to obtain 1D and 2D NMR spectra, using TMS as a reference standard. Coupling constants are reported in Hz, and chemical shifts (*δ*) are given in ppm relative to the solvent signals. The UV-2401PC spectrophotometer (Shimadzu Corp., Kyoto, Japan) was used to record the ultraviolet (UV) spectra. The infrared (IR) spectra were recorded utilizing a Bruker Tensor-27 infrared spectrometer with KBr pellets (Bruker, Billerica, MA, USA). Circular dichroism (CD) spectra were measured using a Chirascan instrument (Agilent 39 Technologies, Santa Clara, CA, USA). Column chromatography methods were performed with a Sephadex LH-20 (Amersham Pharmacia Biotech, Stockholm, Sweden), silica gel (200–300 mesh, Qingdao Ocean, Qingdao, China), medium-pressure liquid chromatography device (RP-18, YMC), and MCI column (75–150 mesh, Mitsubishi Chemical Co., Ltd., Tokyo, Japan).

### 3.2. Plant Materials

The roots of *T. hypoglaucum* were obtained from Kunming in Yunnan Province of China and verified using http://www.theplantlist.org accessed on 3 September 2021. The samples were botanically identified by Wang Anqun from Yunnan Lv Sheng Pharmaceutical Co., Ltd., Kunming, China. Voucher specimens (No. Luo20220201) were preserved at the Kunming Institute of Botany, Chinese Academy of Science.

### 3.3. Extraction and Isolation

Dried and powdered *T. hypoglaucum* (10.0 kg) was extracted with 95% EtOH (50 L × 3) at room temperature and evaporated to produce an EtOH extract (1.25 kg). The crude extract was suspended and dispersed with water, and an appropriate amount of chloroform was added; it was extracted 3 times, and 150 g of the partial chloroform extract was concentrated. The chloroform extract was subjected to a silica gel column using gradient elution with CHCl3/MeOH (80:1→0:1) to provide four fractions (Fr. A–Fr. D). Fr. A (45.0 g) underwent separation via MPLC using gradient elution with MeOH/H_2_O (1:1→1:0), resulting in the production of 5 subfractions. Tripterhyponoid A (50.0 mg) was isolated from Fr. A2 (7.5 g) sequentially using silica gel column chromatography with petroleum–acetone (15:1, *v*/*v*), followed by Sephadex LH-20 column chromatography eluting with CHCl3/CH3OH (1:3, *v*/*v*).

### 3.4. Bacterial Strains and Reagents

*S. aureus* (ATCC 25923, USA300) was procured from the Beijing Baiou Bowei Biotechnology Co., Ltd., Beijing, China. MRSA 003 was obtained from the First People’s Hospital of Zunyi. Additional sources of strains and reagents are elaborated upon in Tables S4 and Table S5. See the Supplementary Materials for the bacterial culture conditions.

### 3.5. Antibacterial Testing

The MICs of tripterhyponoid A and various antibiotics were assessed utilizing the broth microdilution method, as outlined by the Clinical and Laboratory Standards Institute (CLSI, 2021). The MBC was determined by transferring 100 µL of trypticase soy broth (TSB) from each well to TSA plates, which were subsequently incubated at 37 °C for a duration of 24 h [12].

### 3.6. Growth Curve

The growth curve illustrates the colony growth pattern of bacteria cultured under specific environmental conditions and can indicate the impacts of antibacterial compounds on their growth. Briefly, bacterial suspensions of the strains were treated with different concentrations (1/2 MIC, MIC, 2 MIC, 4 MIC) of TA and antibiotics (1/2 MIC, MIC VAN for MRSA003 and USA300; 1/2 MIC, MIC AMP for VRE) at 180 rpm and 37 °C. At designated time intervals (0, 1, 2, 4, 6, 8, 10, 12, 21, 24 h), the OD_600_ of a 0.1 mL solution was measured using a SpectraMax 190 (Molecular Devices, San Jose, CA, USA) [29].

### 3.7. Time-Kill Curve

The time-kill curve illustrates the viability of bacteria when exposed to antibacterial compounds or antibiotics and was measured as previously described [56].

### 3.8. Bacterial Resistance Study

For resistance development by sequential passaging, MRSA003 and VRE in the exponential phase were passaged into new TSB containing TA, VAN, or AMP at concentrations of 1/2 × or 1/4 × MIC. After being incubated at 180 rpm and 37 °C for 24 h, the suspensions were then subjected to the next MIC assay. The process was repeated for 20 generations [57].

### 3.9. USA300 Treatment and Collection

Overnight USA300 cultures were treated with tripterhyponoid A (2 μg/mL) or DMSO for 4 h. Following treatment, the bacterial culture was harvested via centrifugation at 4 °C and 4000 rpm for 10 min and washed twice with PBS, yielding three samples for each group. The transcriptomic data were collected by the Shanghai Majorbio Bio-Pharm Technology Co., Ltd. (Shanghai, China), and the data were analyzed using the online tool https://cloud.majorbio.com/page/tools/ accessed on 1 November 2023 [29]. The raw transcriptomic data have been deposited in NCBI under accession number SUB14345479.

### 3.10. Real-Time Quantitative PCR (RT-qPCR) Validation

Total RNA was isolated using the Bacteria Total RNA Isolation Kit (B518625, Sangon, Shanghai, China), followed by cDNA synthesis (HiScript III RT SuperMix for qPCR (+gDNA wiper) kit, Vazyme, Nanjing, China). RT-qPCR was performed with SYBR Green Master Mix (Vazyme) to measure the mRNA expression levels of target genes. The expression level of 16S mRNA was used for normalization. The primers are listed in Table S6. Relative gene expression was determined using the 2^−∆∆CT^ method [58].

### 3.11. Crystal Violet Assay

The CV assay was employed to evaluate the impacts of TA and antibiotics on biofilm inhibition and mature biofilm eradication, which were measured following a previously described method [12,59].

### 3.12. Qualitative Analysis of Mature Biofilm

A mature MRSA biofilm was formed after 24 h incubation at 37 °C in TSB medium. The medium was discarded, and the biofilm was rinsed 3 times with PBS to remove planktonic bacteria. Tripterhyponoid A, antibiotics, or DMSO were added, and the biofilm was incubated at 37 °C for 12 h. After discarding the medium, the biofilm was washed 3 times with sterile water, fixed with 4% PFA for 1 h, washed again, and dried at room temperature for 30 min. It was then stained with 5-DTAF (10 μg/mL) for 60 min, washed, stained with DAPI (20 μg/mL) for 40 min, washed, and dried. Images were captured using an inverted fluorescence microscope [13].

### 3.13. Imaging of 3D Biofilm

Bacteria were incubated in confocal dishes at 37 °C for 24 h. After removing the medium, the biofilm was washed with PBS, treated or untreated with the compound for 12 h, washed with ddH_2_O, and stained with SYPRO^®^ Ruby for 30 min. Images were taken using a Leica TCS SP8 STED confocal microscope (Leica Microsystems, Wetzlar, Germany) at 488 nm and 450 nm excitation wavelengths [7].

### 3.14. DNA Gel Migration Assay

The interaction between tripterhyponoid A and MRSA genomic DNA was studied using agarose gel electrophoresis analysis. The genomic DNA was extracted using a commercial kit (Vazyme, DC103). The DNA concentration was measured, adjusted to 100 ng/μL, and mixed with tripterhyponoid A at 25 °C for 10 min. The mixtures were electrophoresed on a 0.8% agarose gel, and gel retardation was observed under UV illumination using a BioDoc-It^®^ Imaging System (UVP, Upland, CA, USA) [33,54].

### 3.15. Ultraviolet–Visible (UV–Vis) Spectroscopy and Circular Dichroism (CD) Assay

A total volume of 200 μL of genomic DNA (50 μg/mL) was incubated with tripterhyponoid A at 50, 100, and 200 μg/mL for 10 min. Measurements were taken using a Chirascan V100 CD spectrometer (Applied Photophysics, Britain) with a 1.0 mm quartz cuvette, scanning from 220 to 320 nm at 25 °C and a speed of 10 nm/min [33,54].

### 3.16. Molecular Docking

A molecular docking study using AutoDock 4 was conducted to explore the interaction between *S. aureus* DNA and tripterhyponoid A. The DNA (PDB code: 4xqq) served as the receptor, with the entire nucleic acid chain as the binding site. Different poses of the small-molecule ligands, tripterhyponoid A, were generated by adding hydrogen atoms, and the docking results between the DNA were visualized using PyMOL, LigPlus, and Discovery Studio 2020 [33].

### 3.17. Mouse Skin MRSA Infection Model

This study, approved by the Institutional Animal Care and Use Committee of Yunnan University (No. YNUCARE202210020), used 36 female KM mice (6–8 weeks, 18 ± 2 g) from Kunming Medical University. After a 7-day quarantine in an SPF environment (20–25 °C, 40–70% humidity, 12 h light–dark cycle), the mice were divided into six groups (n = 6): control (CK), model, VAN treatment, and three tripterhyponoid A treatment groups. The mice were injected intraperitoneally with cyclophosphamide (100 mg/kg, 0.1 mL) for 3 days to reduce neutrophils. Following this, the mice were anesthetized with sodium pentobarbital (80 mg/kg) and each given a 6 mm wound. The wounds were inoculated with 25 μL MRSA003 suspension (4 × 10^8^ CFUs/mL). Four hours post-infection, the treatment groups received TA (0.5, 1, or 2 mg/kg) or VAN (2 mg/kg), while the model group received PBS, administered daily. The wound size was measured before each treatment. At the end of the experiment, wound tissue was collected for bacterial load analysis, H&E staining, and ELISA evaluation [56].

### 3.18. Statistical Analysis

All data presented in the graphs are expressed as the mean ± standard deviation (SD). Statistical analyses between groups were conducted using a one-way analysis of variance (ANOVA) followed by a *t*-test. All measurements were performed in at least three replicates. GraphPad Prism version 8.0.1 was utilized for data processing and statistical analysis, where asterisks or octothorpes indicate statistical significance (* *p* < 0.05, ** *p* < 0.01, *** *p* < 0.001; ### *p* < 0.001).

## 4. Conclusions

In this study, a new friedelane triterpenoid (tripterhyponoid A) derived from *T. hypoglaucum* exhibiting anti-MRSA activity was identified. The results from the transcriptomics analysis and RT-qPCR analysis indicated that, under treatment with tripterhyponoid A, the expression levels of genes associated with initial bacterial adhesion to biofilms, capsular polysaccharide synthesis proteins, and virulence factors were reduced. Subsequent experiments involving CV staining and the application of various fluorescent dyes further revealed that it demonstrated significant efficacy in inhibiting and eradicating biofilms formed by the clinically relevant pathogenic bacteria USA300 and MRSA003. Concurrently, the transcriptomics analysis showed that genes related to DNA replication and repair were significantly upregulated, suggesting that tripterhyponoid A caused DNA damage. Subsequent biochemical experiments demonstrated that the combination of tripterhyponoid A and DNA can lead to DNA damage through interactions involving minor groove binding, intercalation binding, and electrostatic interactions. Additionally, its application markedly decreased the bacterial load at the site and modulated inflammatory factors to facilitate infected wound healing in a mouse skin infection model. This study provides a potential lead compound for anti-MRSA applications that acts via multiple mechanisms.

## Figures and Tables

**Table 1:** NMR spectroscopic data for tripterhyponoid A in CD_3_OD.

Position	*δ*_C_*^a^*, Type	*δ*_H_*^b^* (*J* in Hz)	Position	*δ*_C_*^a^*, Type	*δ*_H_*^b^* (*J* in Hz)
1	110.0, CH	6.69, s	16	38.1, CH_2_	1.90, m 1.43, m
2	142.2, C		17	31.6, C	
3	144.7, C		18	45.9, CH	1.58, overlap
4	121.8 C		19	30.9, CH_2_	2.12, overlap 1.38, d, (4.6)
5	125.2, C		20	41.3, C	
6	42.0, CH	3.58, m	21	31.7, CH_2_	1.67, m
7	122.6, CH	6.02, d (6.1)	22	36.0, CH_2_	2.12, overlap 0.92, m
8	151.2, C		23	11.8, CH_3_	2.21, s
9	38.5, C		24	69.2, CH_2_	3.71, dd, (10.3, 4.1) 3.26, t (10.3)
10	142.9, C		25	37.7, CH_3_	1.47, s
11	37.1, CH_2_	2.09, m 1.83, m	26	22.7, CH_3_	1.28, s
12	31.6, CH_2_	2.47, d (15.6) 1.75, d (15.6)	27	19.2, CH_3_	0.75, s
13	39.1, C		28	32.1, CH_3_	1.10, s
14	45.0, C		29	33.3, CH_3_	1.17, s
15	30.1, CH_2_	1.61, overlap	30	182.8, C	

*^a^* Measured at 125 MHz. *^b^* Measured at 500 MHz.

## Data Availability

Data are contained within the article and Supplementary Materials.

## Supplementary Materials

The following supporting information can be downloaded at: https://www.mdpi.com/article/10.3390/molecules30122539/s1, Table S1. Antibacterial activity of tripterhyponoid A and antibiotics against bacteria (μg/mL). Table S2. Functional enrichment of USA300 genes induced and suppressed by tripterhyponoid A. Table S3. Functional enrichment of USA300 upregulated genes. Table S4. Strains used in this study. Table S5. Solvents, reagents, and kits used in this study. Table S6. Primer sequences. Figure S1. ^1^H NMR (500 MHz) spectrum of tripterhyponoid A in CD_3_OD. Figure S2. ^13^C NMR (125 MHz) and DEPT spectra of tripterhyponoid A in CD_3_OD. Figure S3. ^1^H-^1^H COSY spectrum of tripterhyponoid A in CD_3_OD. Figure S4. HSQC spectrum of tripterhyponoid A in CD_3_OD. Figure S5. HMBC spectrum of tripterhyponoid A in CD_3_OD. Figure S6. ROESY spectrum of tripterhyponoid A in CD_3_OD. Figure S7. HRESIMS of tripterhyponoid A. Figure S8. UV spectrum of tripterhyponoid A. Figure S9. IR spectrum of tripterhyponoid A. Figure S10. Experimental ORD tripterhyponoid A. Figure S11. Anti-VRE activity of tripterhyponoid A in vitro. Figure S12. GO enrichment analysis. Figure S13. Heatmap of downregulated genes and the RT-qPCR analysis of MRSA003. Figure S14. Heatmap of upregulated genes of the DNA replication and repair pathways.
