# Supplementary material for: Tripterhyponoid A from Tripterygium hypoglaucum Inhibiting MRSA by Multiple Mechanisms
Source: Molecules. 2025 Jun 10;30(12):2539. doi: 10.3390/molecules30122539 (PMC12195962; doi:10.3390/molecules30122539)
Supplement: Supplementary file 1 [file molecules-30-02539-s001.zip › molecules-3662391-supplementary.pdf]

Support information

Tripterhyponoid A from *Tripterygium hypoglaucum* Inhibiting MRSA by Multiple Mechanisms

Yan-Yan Zhu [1], Qiong Jin [2], Zhao-Jie Wang [1], Mei-Zhen Wei [1], Wen-Biao Zu [1], Zhong-Shun Zhou [1], Bin-Yuan Hu [1], Yun-Li Zhao [1], Xu-Jie Qin [2] and Xiao-Dong Luo [1, 2, *]

[1] Yunnan Characteristic Plant Extraction Laboratory, Key Laboratory of Medicinal Chemistry for Natural Resource, Ministry of Education and Yunnan Province, School of Chemical Science and Technology, Yunnan University, Kunming, 650500, People's Republic of China

[2] State Key Laboratory of Phytochemistry and Plant Resources in West China, Kunming Institute of Botany, Chinese Academy of Sciences, Kunming, 650201, People's Republic of China

*Corresponding Author

E-mail address: xdluo@ynu.edu.cn (X.-D. Luo).

#These authors contributed equally to this work.

Contents

RESULTS and DISCUSSION S4

Table S1. Antibacterial activity of tripterhyponoid A and antibiotics against bacteria (μg/mL) S4

Table S2. Functional enrichment of USA300 genes induced and suppressed by tripterhyponoid A S5

Table S3. Functional enrichment of USA300 up-regulated genes S10

Fig. S1. ^{1}H NMR (500 MHz) spectrum of tripterhyponoid A in CD_3OD. S14

Fig. S2. ^{13}C NMR (125 MHz) and DEPT spectra of tripterhyponoid A in CD_3OD. S14

Fig. S3. ^{1}H-^{1}H COSY spectrum of tripterhyponoid A in CD_3OD. S15

Fig. S4. HSQC spectrum of tripterhyponoid A in CD_3OD. S15

Fig. S5. HMBC spectrum of tripterhyponoid A in CD_3OD. S16

Fig. S6. ROESY spectrum of tripterhyponoid A in CD_3OD. S16

Fig. S7. HRESIMS of tripterhyponoid A. S17

Fig. S8. UV spectrum of tripterhyponoid A. S18

Fig. S9. IR spectrum of tripterhyponoid A. S19

Fig. S10. Experimental ORD tripterhyponoid A. S19

Fig. S11. Anti-VRE activity of tripterhyponoid A *in vitro*. S20

Fig. S12. GO enrichment analysis. S20

Fig. S13. Heatmap of down-regulated genes and the RT-qPCR analysis of MRSA003.. .... S21

Fig. S14. Heatmap of up-regulated genes of the DNA replication and repair related pathways. S21

EXPERIMENTAL SECTION S22

Table S4. Strains used in this study..........S22
Table S5. Solvents, reagents, and kits used in this study. S23
Table S6. Primer sequences. S23

RESULTS and DISCUSSION

Table S1. Antibacterial activity of tripterhyponoid A and antibiotics against bacteria (μg/mL)

Strains	MIC[a]	MBC[a]	MIC[b]	MIC[c]	MIC[d]	MIC[e]	MIC[f]	MIC[g]	MIC[h]	MIC[i]	MIC[J]	MIC[K]
						**G+**						
***S. aureus* ATCC25923**	1	4	1	0.5	1	1	0.5	1	2	0.25	8	0.5
***S. aureus* ATCC43300**	8	-	1	512	> 2048	> 2048	> 2048	2048	128	0.5	8	1024
***S. aureus* 80**	4	-	1	512	256	2048	4	2	512	32	8	512
**MRSA 003**	2	16	1	512	> 2048	> 2048	> 2048	2048	32	128	4	> 2048
**MRSA 011**	2	-	1	512	> 2048	> 2048	> 2048	2048	32	128	4	1024
**MRSA 031**	2	-	3	128	4	8	2	1	16	2	8	16
**USA300**	2	8	1	256	64	128	-	0.5	8	0.5	8	2
***S.epidermidis* CMCC26069**	1	-	2	8	2	2	0.25	0.5	> 2048	64	4	1
***E. faecalis* ATCC32001**	4	-	64	2	512	16	4	16	128	1	4	8
**VRE ATCC51299**	4	32	32	2	1024	16	> 2048	2048	> 2048	2	32	1
***E. durans* SC009**	2	-	1	1024	> 2048	> 2048	> 2048	2048	32	128	4	> 2048
						**G-**						
***P. aeruginosa* Bio-109004**	> 16	-	> 1024	> 1024	> 1024	64	256	128	16	16	64	> 1024
***A. baumnnii* Bio-53272**	> 16	-	512	> 1024	> 1024	512	1024	> 1024	256	8	64	> 1024
E. coli	> 16	-	> 1024	128	2	4	4	> 1024	128	16	64	8

Staphylococcus aureus: *S. aureus*; Methicillin-resistant *Staphylococcus aureus*: MRSA; *Staphylococcus epidermidis*: *S. epidermidis*; *Enterococcus faecalis*: *E. faecalis*;

vancomycin-resistant *Enterococcus*: VRE; *Enterococcus durans*: *E. durans*; *Pseudomonas aeruginosa*: *P. aeruginosa*; *Acinetobacter baumnnii*: *A. baumnnii*; *Escherichia coli*: *E. coli*. MIC: minimal inhibitory concentration; MBC: minimal bactericidal concentration; a, Tripterhyponoid A; b, vancomycin hydrochloride; c, ampicillin sodium; d, cefoxitin; e, cefoperazone sodium; f, azithromycin; g, lincomycin hydrochloride; h, streptomycin sulfate; i, tetracycline hydrochloride; j: chloramphenicol; k, norfloxacin. -: no detected. The experiment was repeated three times.

Table S2. Functional enrichment of USA300 genes induced and suppressed by tripterhyponoid A

Gene name	Log2FC (wth/con)	Padjust	Pathways	Gene description
sarA	-2.19387614	0.0001		global transcriptional regulator SarA
icaR	-1.41672878	＜0.0001		ica operon transcriptional regulator IcaR
fnbA	-1.47935604	＜0.0001		fibronectin-binding protein FnbA
sdrC	-1.08934834	0.0001		MSCRAMM family adhesin SdrC
fnbB	-1.03211851	0.0003		fibronectin-binding protein FnbB
lip2	-2.36576307	＜0.0001		YSIRK domain-containing triacylglycerol lipase Lip2/Geh
mprF	-1.20674755	＜0.0001	TCS/Staphylococcus aureus infection	bifunctional lysylphosphatidylglycerol flippase/synthetase MprF
lrgA	-7.8136719	＜0.0001	TCS	antiholin-like murein hydrolase modulator LrgA
lrgB	-7.77920773	＜0.0001	TCS	antiholin-like protein LrgB
saeR	-3.32289668	＜0.0001	TCS	response regulator transcription factor SaeR
saeS	-3.08238946	＜0. 0001	TCS	two-component system sensor histidine kinase SaeS
uhpT	-3.08039178	＜0.0001	TCS	hexose-6-phosphate:phosphate antiporter
arlR	-2.5365316	＜0.0001	TCS	response regulator transcription factor ArlR
arlS	-2.10870799	＜0.0001	TCS	sensor histidine kinase ArlS
glnA	-1.85765665	＜0.0001	TCS	type I glutamate--ammonia ligase
phoA	-1.78243458	0.0051	TCS	alkaline phosphatase

hptR	-1.73199942	＜0.0001	TCS	response regulator transcription factor
narI	-1.64958337	＜0.0001	TCS	respiratory nitrate reductase subunit gamma
narT	-1.64387692	＜0.0001	TCS	nitrate/nitrite transporter
lytT	-1.54636738	＜0.0001	TCS	response regulator transcription factor LytR
narJ	-1.45164108	＜0.0001	TCS	nitrate reductase molybdenum cofactor assembly chaperone
lytS	-1.40639662	＜0.0001	TCS	sensor histidine kinase
nreC	-1.29162594	＜0.0001	TCS	nitrate respiration regulation response regulator NreC
narG	-1.24834513	＜0.0001	TCS	nitrate reductase subunit alpha
narH	-1.11357787	0.0002	TCS	nitrate reductase subunit beta
wecC	-1.03121972	＜0.0001	TCS	type 8 capsular polysaccharide synthesis protein Cap8O
nisF	-3.19891168	＜0.0001	QS/TCS/ABC transporters	lantibiotic protection ABC transporter ATP-binding subunit
nisE	-3.08189889	＜0.0001	QS/TCS/ABC transporters	lantibiotic immunity ABC transporter MutE/EpiE family permease subunit
epiG	-2.55340891	＜0.0001	QS/TCS/ABC transporters	hypothetical protein
agrA	-3.64975218	＜0.0001	QS/TCS	LytTR family DNA-binding domain-containing protein
agrB	-3.64877252	＜0.0001	QS/TCS	accessory gene regulator AgrB
agrC	-3.48879685	＜0.0001	QS/TCS	GHKL domain-containing protein
nisP	-3.10459354	＜0.0001	QS/TCS	S8 family serine peptidase
kdpE	-2.05527783	0.0001	QS/TCS	response regulator transcription factor
nisB	-1.14493212	0.0001	QS/TCS	lantibiotic dehydratase
nisA	-1.0818712	＜0.0001	QS/TCS	gallidermin/nisin family lantibiotic
oppB	-2.40114167	＜0.0001	QS/ABC transporters	serine protease SplF/ABC transporter permease
oppA	-2.38108916	＜0.0001	QS/ABC transporters	hypothetical protein/peptide ABC transporter substrate-binding protein
oppF	-2.37038876	＜0.0001	QS/ABC transporters	Glu-specific serine endopeptidase SspA/ATP-binding cassette domain-containing protein
oppD	-2.08388542	＜0.0001	QS/ABC transporters	phenol-soluble modulin PSM-alpha-3/ABC transporter ATP-binding protein
oppC	-2.0545505	＜0.0001	QS/ABC transporters	preprotein translocase subunit YajC/ABC transporter permease
agrD	-4.16341023	0.0030	QS/TCS	cyclic lactone autoinducer peptide

psmA3	-4.93182737	0.0040	QS	phenol-soluble modulin PSM-alpha-3
hld	-3.70539176	＜0.0001	QS	delta-haemolysin precurser [Staphylococcus aureus subsp. aureusRN4220]
splA	-3.70422825	＜0.0001	QS	serine protease SplA
splF	-3.47879819	＜0.0001	QS	serine protease SplF
hld	-3.24548215	＜0.0001	QS	delta-lysin family phenol-soluble modulin
sspA	-3.17800973	＜0.0001	QS	Glu-specific serine endopeptidase SspA
splE	-2.96805206	＜0.0001	QS	serine protease SplE
psmB	-2.947108	＜0.0001	QS	beta-class phenol-soluble modulin
splD	-2.70073599	＜0.0001	QS	serine protease SplD
psmA1_2	-2.62558458	＜0.0001	QS	phenol-soluble modulin PSM-alpha-2
psmB	-2.48395528	0.0001	QS	beta-class phenol-soluble modulin
fadD	-2.27482774	＜0.0001	QS	class I adenylate-forming enzyme family protein
comK	-2.0907906	＜0.0001	QS	competence protein ComK
AH5667_RS04510	-2.04055618	0.0004	QS	ABC transporter permease
AH5667_RS00775	-2.03779352	0.0004	QS	ABC transporter permease
AH5667_RS00780	-1.85465101	0.0057	QS	ABC transporter permease
secY2	-1.8123139	＜0.0001	QS	accessory Sec system protein translocase subunit SecY2
secA	-1.62356907	＜0.0001	QS	accessory Sec system translocase SecA2
ddpD	-1.52879782	＜0.0001	QS	ABC transporter ATP-binding protein
AH5667_RS00785	-1.41926999	0.0031	QS	ABC transporter substrate-binding protein
yajC	-1.06188791	＜0.0001	QS	preprotein translocase subunit YajC
artR	-4.2091903	＜0.0001	ABC transporters	amino acid ABC transporter ATP-binding protein
artQ	-3.33410257	＜0.0001	ABC transporters	ABC transporter permease subunit
ganP	-1.86370415	＜0.0001	ABC transporters	sugar ABC transporter permease
mntA	-1.79226198	＜0.0001	ABC transporters	metal ABC transporter ATP-binding protein
ecfT	-1.75516778	＜0.0001	ABC transporters	energy-coupling factor transporter transmembrane component T

phnE	-1.671497	0.0239	ABC transporters	phosphonate ABC transporter%2C permease protein PhnE
metN	-1.66695201	0.0001	ABC transporters	methionine ABC transporter ATP-binding protein
nikB	-1.60756894	＜0.0001	ABC transporters	ABC transporter permease
potB	-1.57658509	0.0110	ABC transporters	ABC transporter permease
mntB	-1.57036094	＜0.0001	ABC transporters	metal ABC transporter permease
nikD	-1.53407402	0.0034	ABC transporters	ABC transporter ATP-binding protein
metI	-1.49133466	＜0.0001	ABC transporters	methionine ABC transporter permease
metI	-1.4353255	0.0003	ABC transporters	methionine ABC transporter permease
msmX	-1.42921312	0.0043	ABC transporters	sn-glycerol-3-phosphate ABC transporter ATP-binding protein UgpC
potA	-1.27851325	0.0009	ABC transporters	ABC transporter ATP-binding protein
metN	-1.27799114	0.0001	ABC transporters	methionine ABC transporter ATP-binding protein
metQ	-1.26654274	＜0.0001	ABC transporters	MetQ/NlpA family ABC transporter substrate-binding protein
fliY	-1.11897148	0.0006	ABC transporters	transporter substrate-binding domain-containing protein
nikA	-1.10705177	＜0. 0001	ABC transporters	staphylopine-dependent metal ABC transporter substrate-binding protein CntA
mntC	-1.10046135	＜0.0001	ABC transporters	metal ABC transporter substrate-binding protein
potD	-2.34052746	＜0.0001	Staphylococcus aureus infection<br>ABC transporters	spermidine/putrescine ABC transporter substrate-binding protein
metQ	-1.45372756	＜0.0001	Staphylococcus aureus infection<br>ABC transporters	dipeptide ABC transporter glycylmethionine-binding lipoprotein
potC	-1.41125358	0.0015	Staphylococcus aureus infection<br>ABC transporters	ABC transporter permease
hlgA	-5.8974821	＜0.0001	Staphylococcus aureus infection	bi-component gamma-hemolysin HlgAB subunit A
hlgC	-4.78837575	＜0.0001	Staphylococcus aureus infection	bi-component gamma-hemolysin HlgCB subunit C
hlgB	-4.59238692	＜0.0001	Staphylococcus aureus infection	bi-component gamma-hemolysin HlgAB/HlgCB subunit B
eap	-3.82768276	＜0.0001	Staphylococcus aureus infection	MAP domain-containing protein
scn	-3.64012827	＜0.0001	Staphylococcus aureus infection	complement inhibitor SCIN-B

fib	-3.48962475	＜0.0001	Staphylococcus aureus infection	complement convertase inhibitor Efb
lukG	-3.22122824	＜0.0001	Staphylococcus aureus infection	bi-component leukocidin LukGH subunit G
aur	-3.02066478	＜0.0001	Staphylococcus aureus infection	zinc metalloproteinase aureolysin
lukH	-2.98500278	＜0.0001	Staphylococcus aureus infection	bi-component leukocidin LukGH subunit H
eap	-2.59484632	＜0.0001	Staphylococcus aureus infection	MAP domain-containing protein
sak	-2.26695647	＜0.0001	Staphylococcus aureus infection	staphylokinase
sbi	-1.99274897	＜0.0001	Staphylococcus aureus infection	immunoglobulin-binding protein Sbi
lukS-PV	-1.64883457	＜0.0001	Staphylococcus aureus infection	Panton-Valentine bi-component leukocidin subunit S
eta	-1.60274996	＜0.0001	Staphylococcus aureus infection	TDT family transporter
ssl5_11	-1.49647677	0.0006	Staphylococcus aureus infection	superantigen-like protein SSL11
clfA	-1.46683262	0.0191	Staphylococcus aureus infection	LPXTG cell wall anchor domain-containing protein, partial [Staphylococcus aureus]
chp	-1.40538502	0.0114	Staphylococcus aureus infection	chemotaxis-inhibiting protein CHIPS
isdA	-1.37949001	0.0019	Staphylococcus aureus infection	LPXTG-anchored heme-scavenging protein IsdA
lukF-PV	-1.23311502	< 0.0001	Staphylococcus aureus infection	Panton-Valentine bi-component leukocidin subunit F
cap8O	-1.03122	< 0.0001	TCS/Biofilm formation	type 8 capsular polysaccharide synthesis protein Cap8O
capN	-1.10328	0.001077		capsular polysaccharide type 5/8 biosynthesis epimerase CapN
cap8B	-1.68092	0.002394		type 8 capsular polysaccharide synthesis protein Cap8B
cap8C	-1.53624	0.008709		type 8 capsular polysaccharide synthesis protein Cap8C
capA	-1.63903	0.015444		capsular polysaccharide type 5/8 biosynthesis protein CapA
cap8E	-1.06048	0.034337	Amino sugar and nucleotide sugar metabolism	type 8 capsular polysaccharide synthesis protein Cap8E
carA	-2.53056	< 0.0001	Pyrimidine metabolism;Alanine, aspartate and glutamate metabolism	carbamoyl phosphate synthase small subunit
carB	-2.35897	< 0.0001	Pyrimidine metabolism;Alanine, aspartate and glutamate metabolism	carbamoyl-phosphate synthase large subunit
pyrR	-2.37792	< 0.0001	Pyrimidine metabolism;Nucleotide metabolism	bifunctional pyr operon transcriptional regulator/uracil phosphoribosyltransferase PyrR
pyrB	-2.63934	< 0.0001	Pyrimidine metabolism;Alanine, aspartate and glutamate metabolism	aspartate carbamoyltransferase catalytic subunit
pyrC	-2.264	< 0.0001	Pyrimidine metabolism	dihydroorotase
pyrD	-1.50437	< 0.0001	Pyrimidine metabolism	quinone-dependent dihydroorotate dehydrogenase

pyrF	-1.4968	< 0.0001	Pyrimidine metabolism	orotidine-5'-phosphate decarboxylase
pyrE	-1.20782	0.0002	Pyrimidine metabolism	orotate phosphoribosyltransferase
arcA	-2.42718	< 0.0001	Arginine biosynthesis	arginine deiminase
arcC	-4.05748	< 0.0001	Arginine biosynthesis;Nitrogen metabolism;Purine metabolism	carbamate kinase
arcD	-4.2446	< 0.0001		arginine-ornithine antiporter

Table S3. Functional enrichment of USA300 up-regulated genes

Gene name	Log2FC (wth/con)	Padjust	Pathways	Gene description
rpsN	3.188352601	< 0.0001	Ribosome	30S ribosomal protein S14
AH5667_RS11710	2.535521124	< 0.0001	Folate biosynthesis	molybdopterin molybdotransferase MoeA
AH5667_RS04350	2.463145138	< 0.0001	Pentose phosphate pathway	glucose-6-phosphate isomerase
rpmG	2.386312194	< 0.0001	Ribosome	50S ribosomal protein L33
moaE	2.31885535	< 0.0001	Sulfur relay system/Folate biosynthesis	molybdenum cofactor biosynthesis protein MoaE
novel0076	2.317922173	0.0004	Pentose phosphate pathway	fructose-bisphosphate aldolase [Staphylococcus schleiferi]
mobA	2.297654695	< 0.0001	Folate biosynthesis	molybdenum cofactor guanylyltransferase MobA
moaB	2.189240964	< 0.0001	Sulfur relay system/Folate biosynthesis	molybdenum cofactor biosynthesis protein B
AH5667_RS13455	2.164694623	< 0.0001	Pentose phosphate pathway	fructose bisphosphate aldolase
queC	2.106580245	< 0.0001	Folate biosynthesis	7-cyano-7-deazaguanine synthase QueC
rplS	2.065149611	< 0.0001	Ribosome	50S ribosomal protein L19
mnmA	2.043419114	< 0.0001	Sulfur relay system	tRNA 2-thiouridine(34) synthase MnmA
moaD	1.984484188	< 0.0001	Sulfur relay system	molybdopterin converting factor subunit 1
moaA	1.855138323	< 0.0001	Folate biosynthesis/Sulfur relay system	GTP 3'%2C8-cyclase MoaA
AH5667_RS05800	1.781015414	< 0.0001	DNA replication	ribonuclease HII

AH5667_RS11520	1.767171076	< 0.0001	Ribosome	type Z 30S ribosomal protein S14
AH5667_RS05285	1.70669486	< 0.0001	Mismatch repair	endonuclease MutS2
dnaX	1.677132039	< 0.0001	DNA replication/Homologous recombination/Mismatch repair	DNA polymerase III subunit gamma/tau
pcrA	1.664585987	< 0.0001	Mismatch repair/Nucleotide excision repair	DNA helicase PcrA
rpmI	1.652201733	< 0.0001	Ribosome	50S ribosomal protein L35
tkt	1.652078409	< 0.0001	Pentose phosphate pathway	transketolase
priA	1.636318942	< 0.0001	Homologous recombination	primosomal protein N'
ligA	1.601745921	< 0.0001	DNA replication/Mismatch repair/Nucleotide excision repair	NAD-dependent DNA ligase LigA
rplU	1.589017108	< 0.0001	Ribosome	50S ribosomal protein L21
rpsT	1.571081463	< 0.0001	Ribosome	30S ribosomal protein S20
recF	1.561079905	< 0.0001	Homologous recombination	DNA replication/repair protein RecF
AH5667_RS09720	1.528177385	< 0.0001	DNA replication/Homologous recombination/Mismatch repair	3'-5' exonuclease
folB	1.523197632	< 0.0001	Folate biosynthesis	dihydroneopterin aldolase
folE2	1.512091356	< 0.0001	Folate biosynthesis	GTP cyclohydrolase FolE2
polA	1.509174829	< 0.0001	DNA replication/Homologous recombination/Nucleotide excision repair	DNA polymerase I
rplX	1.490882865	< 0.0001	Ribosome	50S ribosomal protein L24
rpsP	1.489715251	< 0.0001	Ribosome	30S ribosomal protein S16
rpsR	1.47229561	< 0.0001	Ribosome	30S ribosomal protein S18
AH5667_RS10890	1.444691414	< 0.0001	Pentose phosphate pathway	fructose-bisphosphate aldolase
rpsQ	1.439130568	< 0.0001	Ribosome	30S ribosomal protein S17
iscS	1.424809812	< 0.0001	Sulfur relay system	cysteine desulfurase family protein
AH5667_RS08490	1.396626521	< 0.0001	DNA replication/Homologous recombination/Mismatch repair	DNA polymerase III subunit alpha
AH5667_RS05910	1.392289555	< 0.0001	DNA replication/Homologous recombination/Mismatch repair	DNA polymerase III subunit alpha
AH5667_RS02260	1.381941546	< 0.0001	Ribosome	50S ribosomal protein L25/general stress protein Ctc
recR	1.34066681	< 0.0001	Homologous recombination	recombination mediator RecR

rplW	1.340527027	< 0.0001	Ribosome	50S ribosomal protein L23
holA	1.316872814	< 0.0001	DNA replication/Homologous recombination/Mismatch repair	DNA polymerase III subunit delta
rpmH	1.312730192	< 0.0001	Ribosome	50S ribosomal protein L34
rplV	1.307027075	< 0.0001	Ribosome	50S ribosomal protein L22
AH5667_RS10865	1.283127926	< 0.0001	Ribosome	type B 50S ribosomal protein L31
rpsH	1.28051111	< 0.0001	Ribosome	30S ribosomal protein S8
rpsD	1.272152468	< 0.0001	Ribosome	30S ribosomal protein S4
rpsS	1.267400477	< 0.0001	Ribosome	30S ribosomal protein S19
dnaB	1.252370276	< 0.0001	DNA replication	replicative DNA helicase
rpmC	1.238093194	< 0.0001	Ribosome	50S ribosomal protein L29
rpsF	1.237940627	< 0.0001	Ribosome	30S ribosomal protein S6
rplE	1.216192988	< 0.0001	Ribosome	50S ribosomal protein L5
moeB	1.211078153	< 0.0001	Sulfur relay system	ThiF family adenylyltransferase
pfkA	1.190445405	< 0.0001	Pentose phosphate pathway	6-phosphofructokinase
rplC	1.17666358	< 0.0001	Ribosome	50S ribosomal protein L3
rpsJ	1.176267579	< 0.0001	Ribosome	30S ribosomal protein S10
rpsB	1.167679371	< 0.0001	Ribosome	30S ribosomal protein S2
rplB	1.164640614	< 0.0001	Ribosome	50S ribosomal protein L2
folK	1.151798485	< 0.0001	Folate biosynthesis	2-amino-4-hydroxy-6-hydroxymethyldihydropteridine diphosphokinase
AH5667_RS08905	1.110207253	< 0.0001	Pentose phosphate pathway	transaldolase
rplN	1.099282771	< 0.0001	Ribosome	50S ribosomal protein L14
rpsC	1.085775746	< 0.0001	Ribosome	30S ribosomal protein S3
recA	1.085325675	< 0.0001	Homologous recombination	recombinase RecA
gndA	1.081995996	< 0.0001	Pentose phosphate pathway	NADP-dependent phosphogluconate dehydrogenase
rplF	1.073983245	< 0.0001	Ribosome	50S ribosomal protein L6
uvrC	1.049634442	< 0.0001	Nucleotide excision repair	excinuclease ABC subunit UvrC

rplD	1.020615819	< 0.0001	Ribosome	50S ribosomal protein L4
deoC	1.002668334	< 0.0001	Pentose phosphate pathway	deoxyribose-phosphate aldolase
rplP	1.000636267	< 0.0001	Ribosome	50S ribosomal protein L16

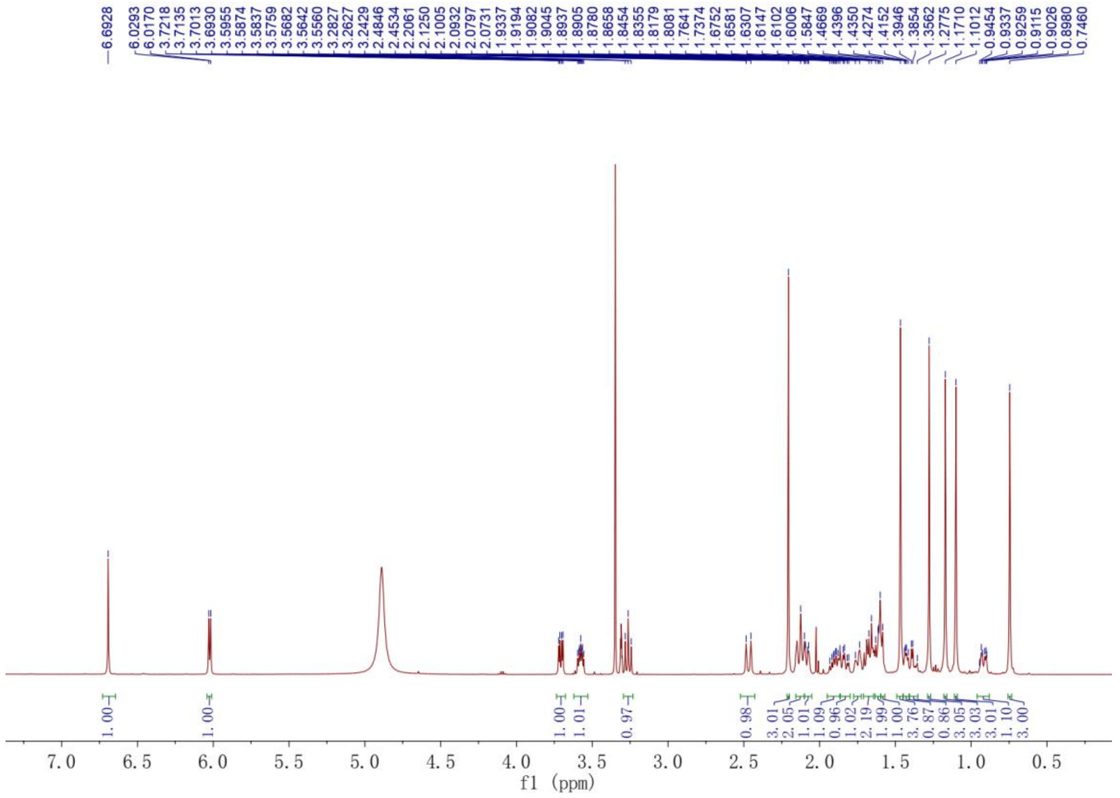

Fig. S1. ^{1}H NMR (500 MHz) spectrum of tripterhyponoid A in CD_3OD.

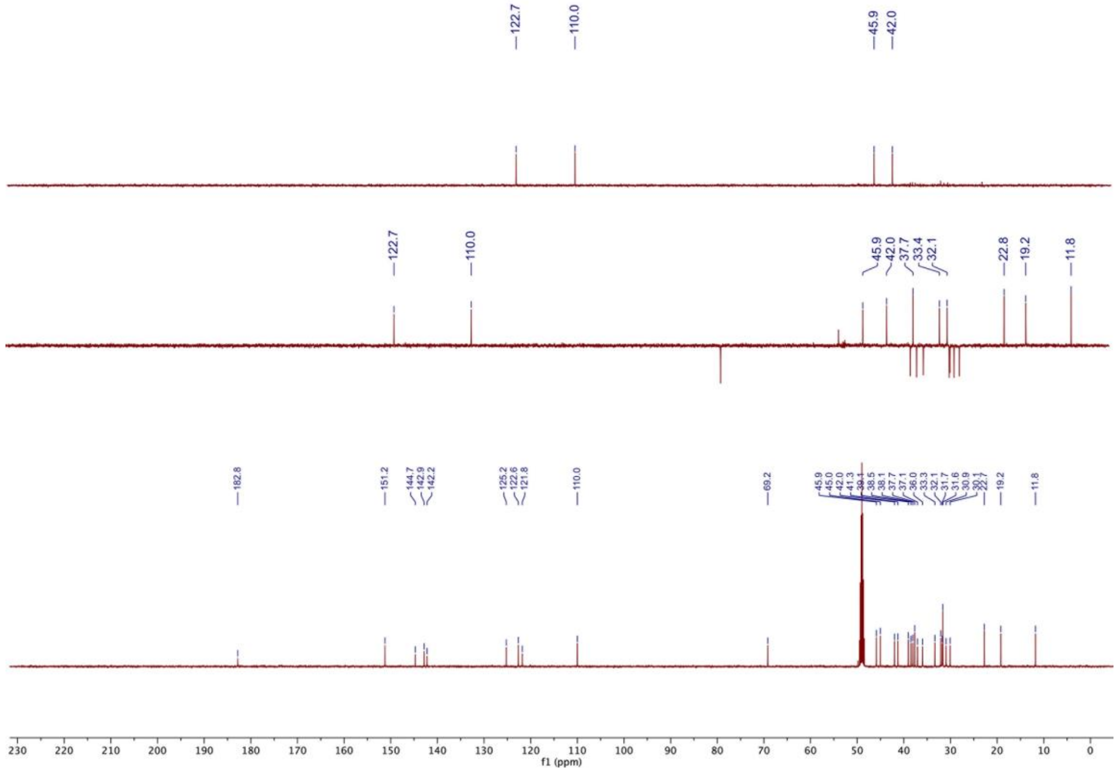

Fig. S2. ^{13}C NMR (125 MHz) and DEPT spectra of tripterhyponoid A in CD_3OD.

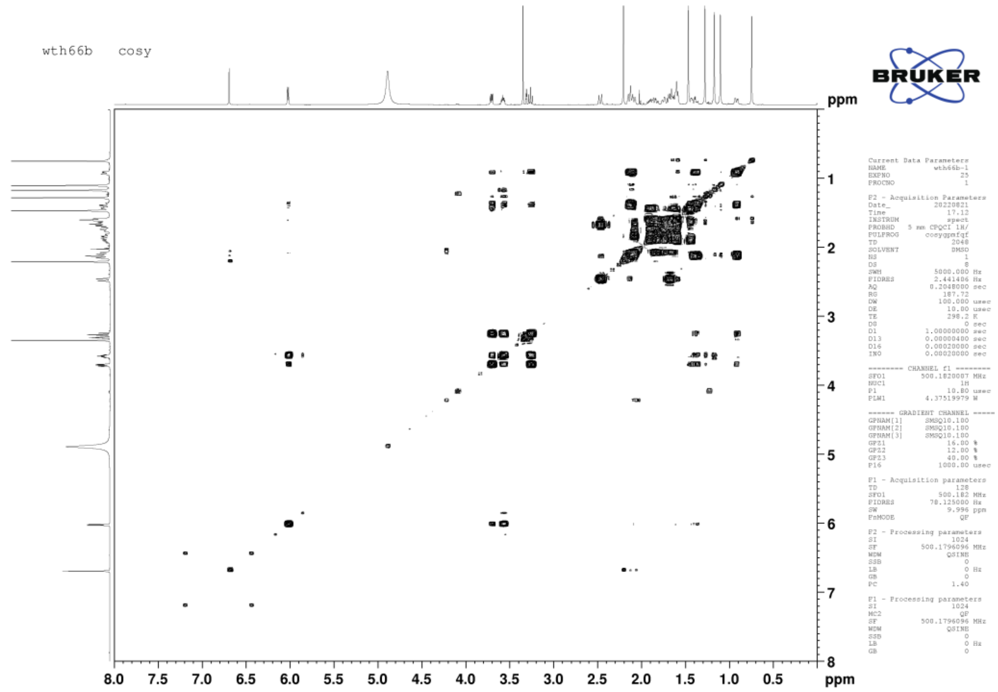

Fig. S3. ^{1}H-^{1}H COSY spectrum of tripterhyponoid A in CD_3OD.

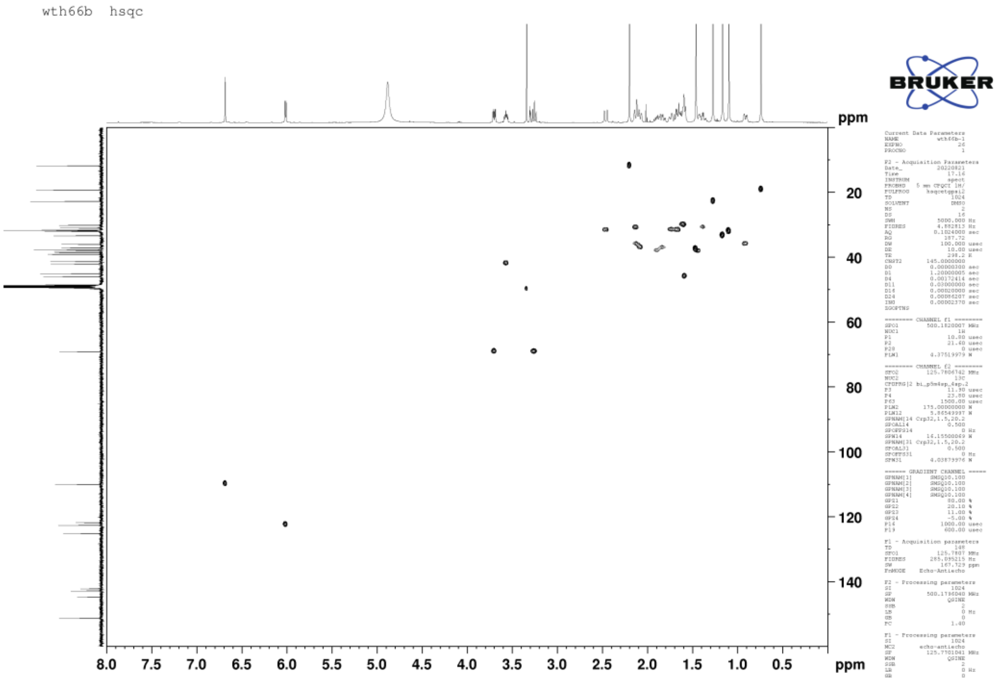

Fig. S4. HSQC spectrum of tripterhyponoid A in CD_3OD.

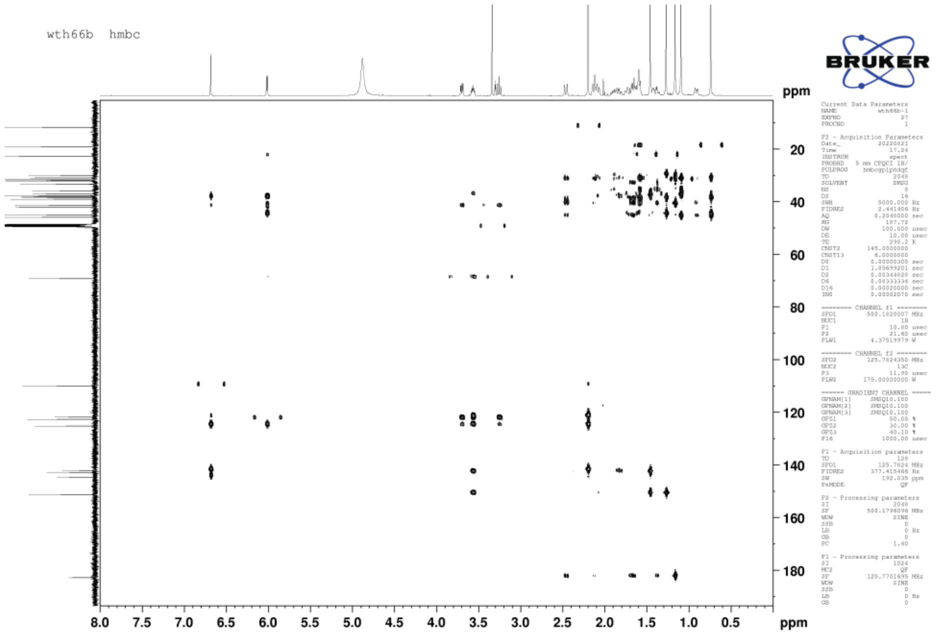

Fig. S5. HMBC spectrum of tripterhyponoid A in CD_3OD.

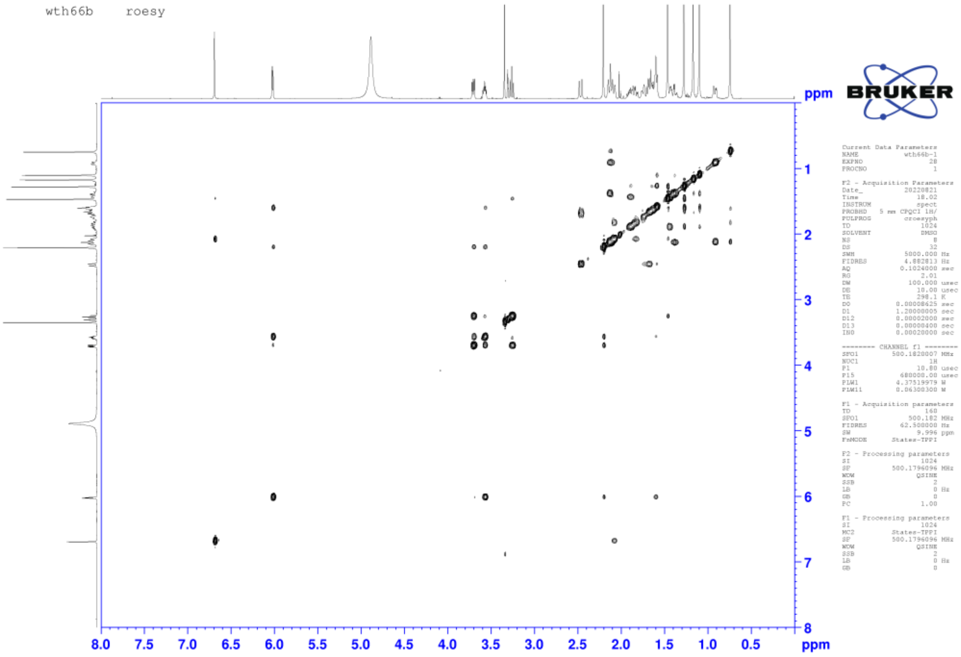

Fig. S6. ROESY spectrum of tripterhyponoid A in CD_3OD.

Qualitative Analysis Report

**Data File**	ZYY-N.d	**Sample Name**	ZYY-N
**Sample Type**	Sample	**Position**	P1-A1
**Instrument Name**	Instrument 1	**User Name**	
**Acq Method**	20220426-liangtong-N.m	**Acquired Time**	5/29/2024 12:54:02 PM (UTC+08:00)
**IRM Calibration Status**	Success	**DA Method**	Default.m
**Comment**			
**Sample Group**		**Info.**	
**Stream Name**	LC 1	**Acquisition Time (Local)**	5/29/2024 12:54:02 PM (UTC+08:00)
**Acquisition SW Version**	6200 series TOF/6500 series Q-TOF B.09.00 (B9044.0)	**QTOF Driver Version**	8.00.00
**QTOF Firmware Version**	25.723	**Tune Mass Range Max.**	3200

Spectra

Fragmentor Voltage	Collision Energy	Ionization Mode
120	0	ESI

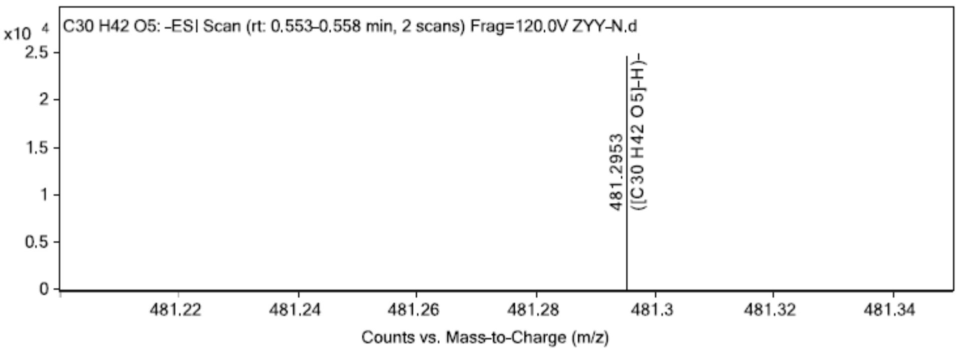

Spectrum Identification Results: - Scan (rt: 0.553-0.558 min) (ZYY-N.d)

Best	ID Sourc	Mass (MFG	Score (MFG	Formula	m/z	DB	Mass	Species	Scor	Diff (ppm	Diff (abs. ppm	Diff (mDa	Score (DB
✓	**MFG**	**482.3032**	**38.31**	**C30 H42 O5**	**481.2953**	**10**	**482.299**	**(M-H)-**	**19.16**	**8.43**	**8.43**	**4.07**	**0**

m/z	Score (iso. abund	Score (mass	Score (MS	Score (MFG	Score (iso. spacing	Heigh	Ion Formul
481.2953	5.91	47.21	38.31	38.31	59.39	28424.9	C30 H41 O5

Height (Calc)	Height Sum% (Cal	Height % (Calc	m/z (Calc	Diff (mDa	Heigh	Height	Height Sum	m/z	Diff (ppm
28424.9	71.7	100	481.2959	0.7	24623.7	100	62.1	481.295	1.37
9411.3	23.7	33.1	482.2993	1.1	8335.4	33.9	21	482.298	2.21
1800.2	4.5	6.3	483.3023	20.4	6677.3	27.1	16.8	483.282	42.17

--- End Of Report ---

Fig. S7. HRESIMS of tripterhyponoid A.

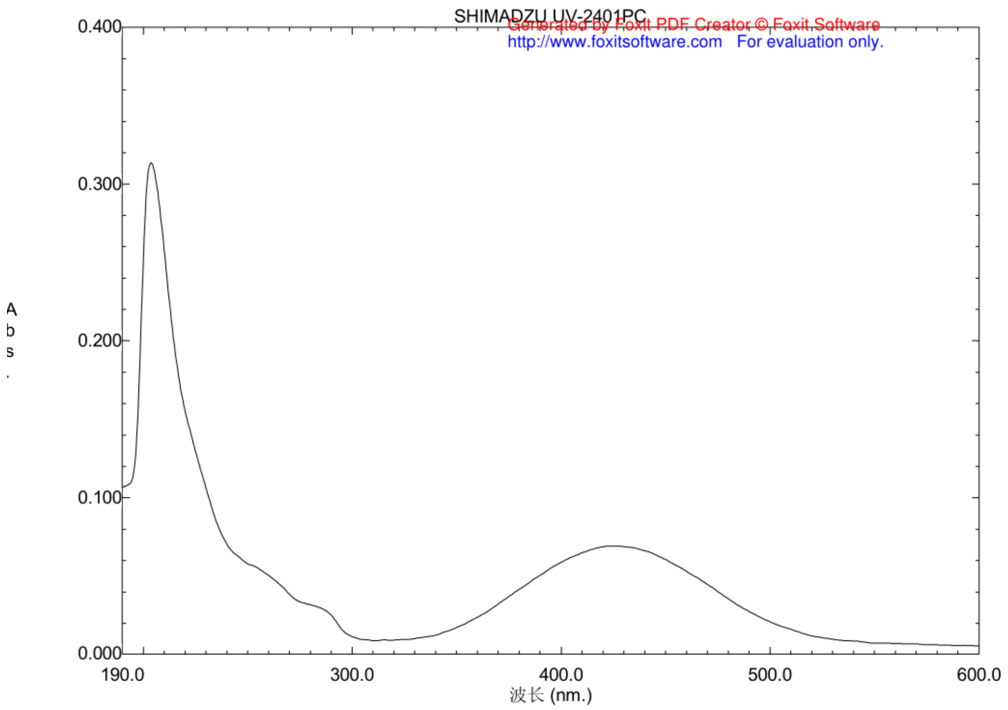

文件名: WTH-66B

WTH-66B———

样品浓度：0.0057毫克/毫升
溶剂：甲醇

创建于: 16:41 22-11-05
数据: 原始

测量模式: Abs.
扫描速度: 中速
狭缝: 5.0
采样间隔: 0.5

否.	波长 (nm.)	Abs.
1	203.00	0.3115
2	249.50	0.0580
3	278.50	0.0320
4	426.00	0.0689

Fig. S8. UV spectrum of tripterhyponoid A.

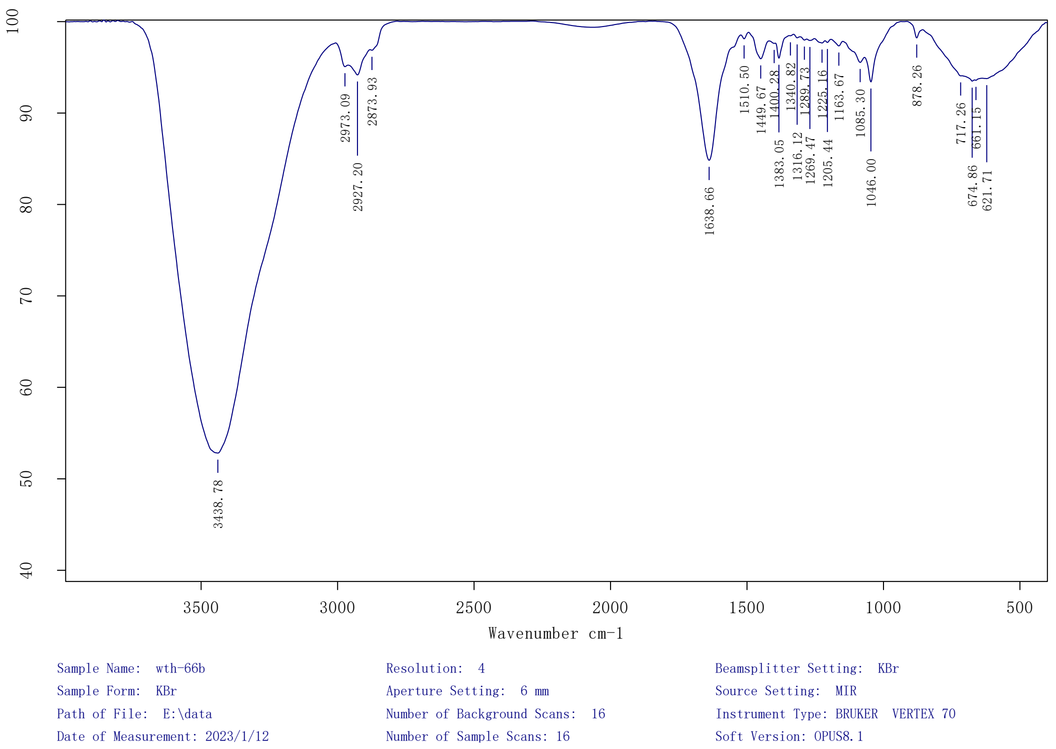

Fig. S9. IR spectrum of tripterhyponoid A.

Rudolph Research Analytical

This sample was measured on an Autopol VI, Serial #91058
Manufactured by Rudolph Research Analytical, Hackettstown, NJ, USA.

Measurement Date : Wednesday, 19-OCT-2022

Set Temperature : 20.0

Time Delay : Disabled

Delay between Measurement : Disabled

n	Average	Std.Dev.	% RSD	Maximum	Minimum
5	-110.00	0.50	-0.45	-109.52	-110.71

S.No	Sample ID	Time	Result	Scale	OR °Arc	WLG.nm	Lg.mm	Conc.g/100ml	Temp.
1	WTH-66B	12:13:06 PM	-109.52	SR	-0.184	589	100.00	0.168	20.5
2	WTH-66B	12:13:12 PM	-109.52	SR	-0.184	589	100.00	0.168	20.4
3	WTH-66B	12:13:19 PM	-110.12	SR	-0.185	589	100.00	0.168	20.4
4	WTH-66B	12:13:25 PM	-110.71	SR	-0.186	589	100.00	0.168	20.3
5	WTH-66B	12:13:31 PM	-110.12	SR	-0.185	589	100.00	0.168	20.3

Fig. S10. Experimental ORD tripterhyponoid A.

ECD calculations

The ECD calculations of tripterhyponoid A were performed by Gaussian 16. The conformational analysis was performed by CONFLEX 8B software using MMFF94s molecular force field with a search limit of 1.0 kcal/mol to yield nine conformers (Supplementary data), respectively. These initial structures were optimized *via* the Density Functional Theory (DFT) at the B3LYP/6-31+G(d) level in gas phase. The optimized conformations were used for ECD calculations by the Time Dependent DFT

(TDDFT) at the B3LYP/6-311+G (d,p) level. The calculated ECD curves were generated by SpecDis (version 1.71).

No.	Distribution	Relative Energy	No.	Distribution	Relative Energy
Conformer	18.9520	0.0000	Conformer	6.3528	0.6476
Conformer	14.5635	0.1561	Conformer	6.1635	0.6655
Conformer	11.7540	0.2830	Conformer	5.6601	0.7160
Conformer	10.7116	0.3381	Conformer	5.2339	0.7624
Conformer	7.2997	0.5653			

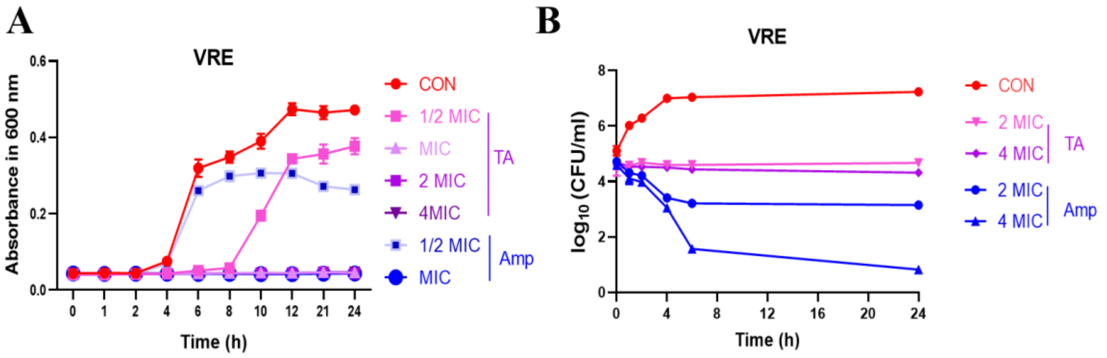

Fig. S11. Anti-VRE activity of tripterhyponoid A *in vitro*. (A) Growth curve of tripterhyponoid A against VRE. (B) Time-kill curve of tripterhyponoid A against VRE.

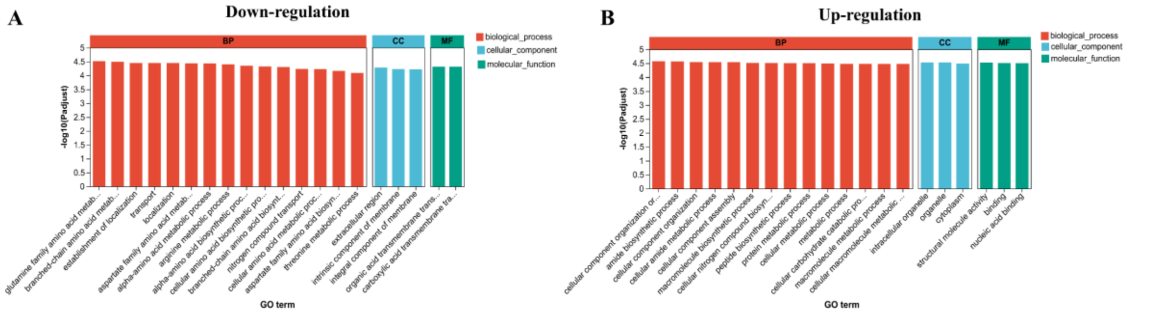

Fig. S12. GO enrichment analysis. (A) GO enrichment analysis of down-regulation genes. (B) GO enrichment analysis of up-regulation genes.

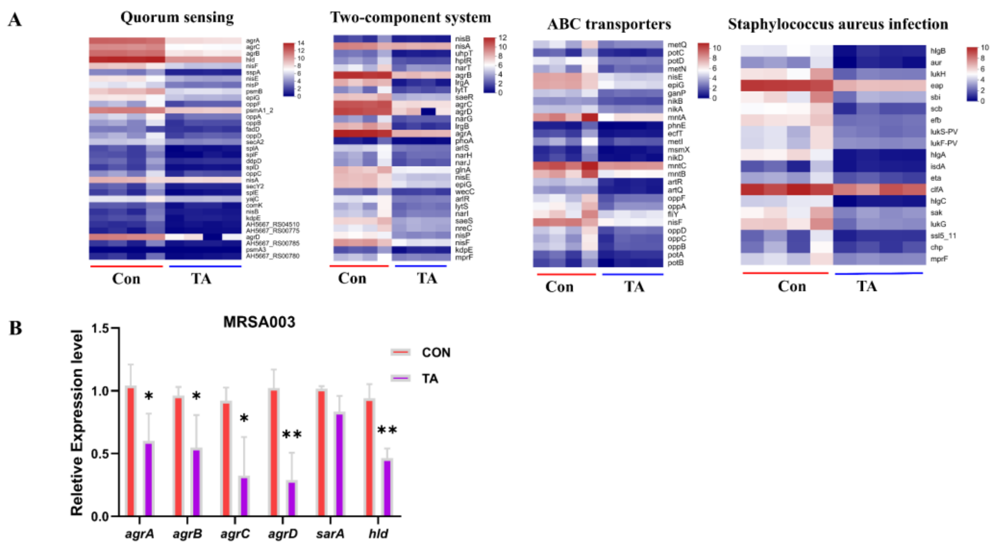

Fig. S13. Heatmap of down-regulated genes and the RT-qPCR analysis of MRSA003. (A) Heatmap of down-regulated genes of the biofilnm and virlences related pathways. (B) The RT-qPCR analysis of genes involved in the QS system. Values are mean ± SD. *p < 0.05 and **p < 0.01 *vs.* CON.

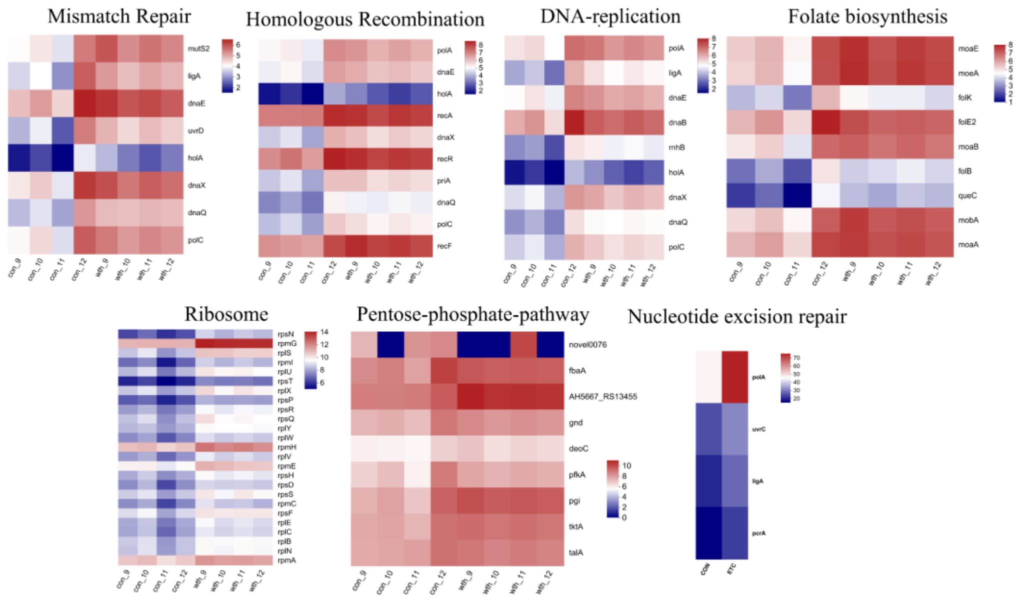

Fig. S14. Heatmap of up-regulated genes of the DNA replication and repair related pathways.

EXPERIMENTAL SECTION

Bacterial Strains and Reagents. All strain sources are detailed in **Table S4**, and the identification of all bacterial strains was validated through 16S ribosomal DNA sequencing. The bacteria were cultured on standard Tryptic Soy agar plates (TSA) and in Tryptic Soy Broth (TSB) at a temperature of 37 °C, with storage at 4 °C maintained throughout the duration of the study, unless specified otherwise. Sources of all reagents and kits are listed in **Table S5**.

Table S4. Strains used in this study.

Strains	Description	Source/Reference
Staphylococcus aureus ATCC 25923 (MSSA)	Standard strain	Biobw
Staphylococcus aureus ATCC 43300 (MRSA)	Standard strain	ATCC
Staphylococcus aureus Bio-114866 (USA300) (MRSA)	Standard strain	Biobw
Staphylococcus aureus 80 (MRSA)	Clinical strain	Lab stock
MRSA 003 (MRSA)	Clinical strain	Lab stock
MRSA 011 (MRSA)	Clinical strain	Lab stock
MRSA 031 (MRSA)	Clinical strain	Lab stock
Staphylococcus epidermidis CMCC 26069	Standard strain	CMCC
Enterococcus faecalis	Clinical strain	Lab stock
Vancomycin-resistant *Enterococcus* ATCC 51299	Standard strain	Biobw
Enterococcus durans SC009	Clinical strain	Lab stock
Pseudomonas Aeruginosa Bio-109004	Commercial strain	Biobw
Acinetobacter baumnnii Bio-53272	Commercial strain	Biobw
Escherichia coli	Clinical strain	Lab stock

MSSA, methicillin-susceptible *Staphylococcus aureus*; MRSA, methicillin-resistant Staphylococcus aureus; ATCC, American Type Culture Collection; CMCC, National Center for Medical Culture Collections; Biobw, beijing baiou bowei biotechnology Co., Ltd. The strains labeled as Lab stock were from the First People's Hospital of Zunyi

Table S5. Solvents, reagents, and kits used in this study.

Solvents/reagents/kits	Brand	Product number
FastPure Bacteria DNA Isolation Mini Kit	Nanjing vazyme Biotechnology Co., Ltd	DC103-01
DNase free & RNase free water	Beijing Coolaber Technology Co., Ltd	SL2250
SYTOX Green Nucleic Acid Stain	Shanghai Maokang Biotechnology Co., Ltd	MX4228
Hoechst 33342 Stain solution	Solarbio science & technology (Bei Jing) Co., Ltd	C0031
FilmTracer™ SYPRO® Ruby Biofilm Matrix Stain	Thermo Fisher Scientific Inc	F10318
SYTO 9 Green Fluorescent Nucleic Acid Stain	Shanghai Maokang Biotechnology Co., Ltd	MX4229
Propidium iodide	Shanghai Macklin Biochemical Technology Co., Ltd	P81650
Agar	Beijing Coolaber Technology Co., Ltd	CA1331
Tryptic Soy Broth	Guangdong Huankai Microbial Sci. & Tech. Co., Ltd.	24051
Agarose	TransGen Biotech Co., Ltd. Beijing	GS201-01
Ethanol	Xilong Scientific Co., Ltd. Sichuan	-
Isopropanol	Chongqing ChuanDong Chemical Co., Ltd.	-
$CHCl_3$	Chongqing ChuanDong Chemical Co., Ltd.	-
Lysozyme	Nanjing vazyme Biotechnology Co., Ltd	DE103-01
HiScript III RT SuperMix for qPCR (+gDNA wiper)	Nanjing vazyme Biotechnology Co., Ltd	R323-01
Taq Pro Universal SYBR qPCR Master Mix	Nanjing vazyme Biotechnology Co., Ltd	Q712-02
Bacteria Total RNA Isolation Kit	Sangon biotech shanghai co. ltd	B518625-0050

Table S6. Primer sequences.

Gene	Primer (5' - 3')	Primer (5' - 3')	Length (bp)
16S	ACTCCTACGGGAGGCAGCAG	ATTACCGCGGCTGCTGG	197
agrA	AAGCCTATGGAAATTGCCCTC	ATGCTTACGAATTTCACTGCCTA	150
agrB	AAATTGACCAGTTTGCCACGTA	GCTAAGACCTGCATCCCTAATCG	91
agrC	GATGACCCTATCATTCGCGTTG	CCTAAACCACGACCTTCACCT	146
agrD	AACATTGGTAACATCGCAGCTT	CGTGTAATTGTGTTAATTCTTTTGGT	85
sarA	ATTCTTTCTCTTTGTTTTCGCTGA	TTGTTATCAATGGTCACTTATGCTG	118
hld	TGAATTTGTTCACTGTGTCGAT	GAGTGATTTCAATGGCACAAGA	81

Tripterhyponoid A: amorphous powder; $[\alpha]^{20}_{D}$ -110.0 (*c* 0.17, CH_3OH); UV (CH_3OH) λ_{max} (log ε) 426 (4.77), 278 (4.43), 249 (4.69), 203 (5.42) nm; IR (KBr) ν_{max} 3439, 2927, 1639, 1450, 1383, 1046 cm^{-1}; CD (CH_3OH) λ_{max} ($\Delta\varepsilon$) 208 (-9.08) nm; ^{1}H and ^{13}C NMR spectral data, see **Table 1**; HRESIMS *m/z* 481.2953 $[M - H]^-$ (calcd for $C_{30}H_{41}O_5$, 481.2959).

H&E staining. The tissue of the skin wounds was fixed in 10% buffered formaldehyde, blocked in paraffin, and transversely sectioned with a microtome (5 mm) for the confection of histological slides. The general analysis, number of blood vessels, and sebaceous glands were assessed by hematoxylin and eosin (H&E) staining.

ELISA evaluation. Skin tissues were weighed and homogenized using a lysis buffer. After grinding the tissues, the supernatants were collected and used to measure the expression levels of the IL-6 and IL-10 inflammatory cytokines. The ProcartaPlex Mouse Cytokine Panel was used to measure cytokine levels based on the manufacturer's protocols.
